# Integrative analysis of bulk and single-cell RNA sequencing data reveals increased arachidonic acid metabolism in osteoarthritic chondrocytes

**DOI:** 10.3389/fmed.2025.1552029

**Published:** 2025-05-09

**Authors:** Kan Wu, Zhaoqian Zhong, Li Chen, Haihua Luo, Aolin Jiang, Linlin Tao, Yong Jiang

**Affiliations:** ^1^Guangdong Provincial Key Laboratory of Proteomics, State Key Laboratory of Organ Failure Research, Department of Pathophysiology, School of Basic Medical Sciences, Southern Medical University, Guangzhou, China; ^2^The First Affiliated Hospital of USTC, Division of Life Sciences and Medicine, University of Science and Technology of China, Hefei, Anhui, China

**Keywords:** MIF, arachidonic acid, osteoarthritis, chondrocyte, bulk RNA sequencing, single-cell RNA sequencing

## Abstract

**Background:**

Abnormal lipid metabolism in chondrocytes, especially arachidonic acid (AA) metabolism, has attracted considerable attention in promoting osteoarthritis (OA) progression. However, the metabolic regulation of chondrocytes in OA remains to be investigated.

**Methods:**

Bulk RNA sequencing (RNA-seq) data and single-cell RNA sequencing (scRNA-seq) data of human knee cartilage were downloaded from public databases. Gene set variation analysis (GSVA) and weighted correlation network analysis (WGCNA) were used to explore functional regulation and gene expression characterization. A reference gene set from the Kyoto Encyclopedia of Genes and Genomes (KEGG) database was used to validate metabolic changes. CellChat analysis was performed to investigate the communication among osteoarthritic chondrocytes. Human immortalized chondrocytes were stimulated with macrophage migration inhibitory factor (MIF), then quantitative real-time PCR (qPCR) and western blot (WB) analysis were used to detect transcription or translation levels of genes. Enzyme linked immunosorbent assay (ELISA) was used to measure AA content. Cartilage from OA patients was collected for immunohistochemistry (IHC) to validate protein expression.

**Results:**

Functional analysis revealed significant activation of the AA metabolic pathway was significantly enriched in the cluster of proliferative chondrocytes (ProCs). CellChat analysis indicated that the incoming signals of MIF increased in ProCs, and the expressions of extracellular signal-regulated kinase (ERK) and phospholipase A2 group IVA (PLA2G4A) were upregulated. Moreover, functional analysis showed that the ERK pathway was enriched in ProCs. Cell experiments indicated MIF stimulation elevated phospho-ERK (p-ERK) and PLA2G4A expression and AA content. IHC showed p-ERK and PLA2G4A were significantly activated in OA cartilage. MIF also upregulated interleukin 1β (IL1B) and matrix metalloproteinase 13 (MMP13) expression.

**Conclusion:**

Our study shows that MIF stimulation of chondrocytes can activate the ERK/PLA2G4A signaling pathway and increase AA production. ProCs located in the proliferative layer of cartilage are likely the main cells executing this mechanism. Therefore, targeting and inhibiting MIF signaling in chondrocytes, especially in ProCs, could be a novel strategy for the prevention and treatment of OA.

## Introduction

Osteoarthritis (OA), a chronic inflammatory disease that affects the joint tissue, is the leading cause of physical disability in adults (1). OA involves various pathological changes, including structural degradation and dysfunction of articular cartilage (2, 3). In recent years, several studies have indicated that abnormal chondrocyte metabolism resulting from inflammatory microenvironment changes is an important factor in cartilage degradation (4). The metabolism of healthy chondrocytes regulates a delicate balance between the breakdown and production of the cartilage matrix, which is crucial for facilitating the self-repair of cartilage to maintain structure integrity (5). However, when this balance is disturbed, such as dysregulation of amino acid metabolism, dysfunction of mitochondrial energy metabolism, and disturbances of lipid metabolism, it can facilitate the progression of OA (6–8). Therefore, it is important to investigate the metabolic regulatory mechanisms related to pro-inflammatory processes in chondrocytes.

Recent studies have revealed that the inflammatory cascade in chondrocytes of patients with OA is linked to the metabolism of lipid mediators, with arachidonic acid (AA) being a prominent pro-inflammatory mediator (9). It has been demonstrated that metabolites of AA, such as prostaglandins and leukotrienes, play essential roles not only in acute inflammation, such as sepsis, but also contribute to the inflammatory response of OA. However, there is currently limited research on the specific characteristics of AA metabolism in chondrocytes of OA. Phospholipase A2 group IVA (PLA2G4A) is recognized as a key regulator in AA metabolism that initiates AA metabolism. It has been reported that macrophage migration inhibitory factor (MIF)-induced activation of extracellular signal-regulated kinase (ERK) signaling upregulates the expression of PLA2G4A in fibroblast cell line, NIH/3T3., thereby promoting AA metabolism (10). However, little has been reported on the expression and regulation of this pathway in osteoarthritic chondrocytes. In addition, osteoarthritic chondrocytes have been classified into various subtypes, including proliferative chondrocytes (ProCs), pre-hypertrophic chondrocytes (preHTCs), hypertrophic chondrocytes (HTCs) chondrogenic progenitor cells (CPCs), homeostatic chondrocytes (HomCs), fibrocartilage chondrocytes (FCs), regulatory chondrocytes (RegCs), and so on (11). However, metabolic regulation at the level of OA chondrocyte subpopulations is currently unknown. Additionally, previous bioinformatics analyses on OA primarily focused on identifying hub genes in synovial and cartilage tissues (12), as well as identifying key pathways associated with inflammation (13). However, the role of metabolic regulation in OA has been rarely analyzed. Thus, it is necessary to investigate the metabolic signatures associated with the osteoarthritic progression by integrating and analyzing RNA sequencing (RNA-seq) data. Due to the heterogeneity of OA, there are variations in clinical and biochemical characteristics of OA patients (14), leading to significant uncertainties in the outcomes of analysis. Therefore, the analysis of a substantial number of samples is an effective way to alleviate such uncertainties (15). Collectively, integrated analysis of multiple OA-related RNA-seq datasets is important for exploring metabolic alterations in chondrocytes of patients with OA.

In this study, we conducted an in-depth analysis of bulk and single-cell RNA sequencing (scRNA-seq) data and combined the analysis with experiments to explore the mechanisms of metabolic regulation in chondrocytes from OA patients. We found that MIF incoming signals increased in arthritic chondrocyte ProCs. We also found that MIF-mediated activation of the ERK/PLA2G4A pathway led to increased AA production in chondrocytes. In conclusion, our findings provide new ideas for the prevention and treatment of OA.

## Materials and methods

### Data sources and pre-processing

The datasets GSE114007 (16) and GSE16850 (17) were downloaded from the Gene Expression Omnibus (GEO) database.^1^ In the dataset GSE114007, sequencing of 18 samples was performed using the Illumina HiSeq 4000 platform, and 20 samples were sequenced using the Illumina NextSeq 500. The dataset GSE168505, which consists of 7 samples, was sequenced using the Illumina HiSeq 2500 platform. The dataset E-MTAB-7313 was downloaded from the European Bioinformatics Institute (EBI) database^2^ and was sequenced using the Illumina HiSeq 4000 platform. Collectively, these datasets encompass 99 samples from four sequencing platforms, including 48 normal samples and 51 OA samples (Supplementary Table 1). We downloaded the FASTQ files by Aspera and trimmed the reads using TrimGalore. Filtered reads were aligned to the GRCh38 reference genome by HISAT2. After obtaining the count matrices of these datasets, TPM normalization was applied to normalize the read counts of the mRNA population for each sample. Subsequently, we merged these three datasets and used the removeBatchEffect function of the linear models for microarray data (limma) in the R software package (18) to remove batch effects.

Metabolism-related genes were sourced from the Kyoto Encyclopedia of Genes and Genomes (KEGG) database.^3^ We filtered out 11 pathways associated with human metabolism and, subsequently organized the corresponding genes from these pathways. After eliminating duplicated genes, a total of 1667 metabolism-related genes were obtained for subsequent analyses.

### Screening for differentially expressed genes (DEGs)

The limma R package is a differential expression screening tool based on generalized linear models. Here, we used the limma package (version 3.54.2) to conduct differential analysis on the expression profiling dataset, resulting in the determination of significance for differences in each gene. We screened DEGs by applying the following criteria: an absolute value of log2 fold change (log2FC) > 1, *p* < 0.05 to control for false discoveries. Subsequently, we plotted a volcano plot and a heat map to represent the expression of DEGs.

### Functional enrichment analysis

We used org.Hs.eg.db R package (version 3.1.0) (19) to transform the gene symbol into the Entrez ID. We performed gene ontology (GO) and KEGG pathway enrichment analysis with DEGs using the clusterProfiler R package (version 3.14.3) (20) (parameters: minGSSize = 5; maxGSSize = 5000; *p*AdjustMethod = “BH”; qvalueCutoff = 0.05).

We calculated the enrichment score for each sample in the referenced gene set of KEGG using the GSVA R package (version 1.40.1) (21). We combined all samples to obtain the matrix of enrichment scores and analyzed the differences using the limma R package. In addition, Gene set enrichment analysis (GSEA) (22) was used to analyze the changes in metabolic pathway regulation in patients with OA.

### Protein-protein interaction (PPI) network

PPI network analysis, a commonly employed method for discerning relationships among proteins, was created using the STRING database (version 11.5) (23). PPI network was downloaded from the STRING database, and was visualized using the Cytoscape software (Version: 3.9.1). We conducted the enrichment analysis of the genes in the PPI network using the ClueGO plugin (Version v2.5.9) (24) with default settings.

### Weighted gene co-expression network analysis (WGCNA)

WGCNA package (version 1.69) (25) was employed to explore expression modules related to OA. We identified correlations between gene modules and OA/sex/age using WGCNA with default parameters. Gene connectivity was measured by the absolute value of Pearson correlation. It was used to filter the central genes of the module with gene significance (GS) ≥ 0.4 and module membership (MM) ≥ 0.8.

### Analysis of scRNA-seq data

The single-cell dataset from GSE169454 contains samples from three normal cartilage samples and four OA cartilage samples. To avoid introducing additional feature filtering processes and to ensure sufficient cell numbers for subsequent analyses, we downloaded the filtered data from the dataset. We created Seurat objects using the “CreateSeuratObject” function from the Seurat package (version 4.9.1) (26). Harmony (27), an algorithm for fast and accurate integration of scRNA-seq data, was used to remove batch effects among samples. The “PercentageFeatureSet” function and the “pattern” parameter (mitochondrial: “^MT−”; rRNA: “^RPL| ^RPS”) was employed to assess rRNA and mitochondrial content. In the first dimensional reduction step, the number of PCA was set to 30. In the second dimensional reduction step, the “dims” parameter was set to 1:30, and the “resolution” parameter was set to 0.1. In addition, Marker genes for each cluster were screened by the “FindAllMarkers” function with default settings, and the cell types were annotated with reference to the Ji et al. (11) study. Based on the top 200 marker genes for each cell cluster, GO and KEGG enrichment analyses were performed using the clusterProfiler R package (28). The scGSVA package (version 0.0.11) (21) was used to calculate the normalized enrichment score (NES) of metabolism-related pathways for each cell cluster.

### Analysis of intercellular communication

Exploring interactions and communication among cell types through ligand-receptor pairs helped us to better understand complex systems biology networks. Therefore, we used the CellChat (v1.1.3) (29) package to infer cell-cell interactions and communication according to the expression of known ligand-receptor pairs. We used the “netVisual_circle” and “netVisual_heatmap” functions in the CellChat package to visualize the communication among cell types and used the “netAnalysis_signalingRole_scatter” and “netAnalysis_signalingChanges_scatter” functions to identify significant ligand-receptor pairs in each cluster.

### Cartilage samples collection

The experiments involving human samples in this study were approved by the Ethics Committee of the Third Affiliated Hospital of Southern Medical University. The cartilage samples for both the osteoarthritis (OA) group and the normal group were obtained from OA patients. In the OA group, the cartilage was collected from the diseased knee joint cartilage, while the cartilage distant from the lesion area, which was macroscopically intact, was taken as the normal group. A total of six cartilage samples were obtained from three patients who underwent total knee arthroplasty (TKA), with one normal cartilage sample and one diseased cartilage sample isolated from each patient (Supplementary Table 2). Right after excision, the samples were fixed in 4% paraformaldehyde for subsequent processing.

### Histological and immunohistochemistry (IHC) assay

All of the samples were fixed in 4% paraformaldehyde for 24 h immediately after isolation. After being decalcified with 10% EDTA for 4 weeks, these samples were embedded in paraffin and serially sectioned (thickness = 2 μm) in the sagittal plane. Sections were stained using hematoxylin and eosin (H&E staining) or Safranin-O and Fast Green staining kit.

For immunochemistry staining, the sections were dewaxed, hydrated, and then immersed in 3% hydrogen peroxide for 10 min to block endogenous peroxidase. Antigen retrieval was performed using citrate buffer at 60°C for 3 min. Next, the sections were divided into two subsets. Each subset was blocked in 5% goat serum for 30 min. Then, one subset was incubated with the primary antibody anti-PLA2G4A (1:500, MK62414, Abmart, China) and the other with anti-phospho-ERK1/2 (1:500, T40072, Abmart, China) at 37°C for 1 h. After being washed five times with PBS, the sections were incubated with a secondary antibody at 37°C for 30 min. Then the sections were incubated for 2 min using the DAB detection system (Gene Tech, Shanghai, China) and the signals were observed under a microscope. Finally, we used ImageJ software to calculate the percentage of positively-stained cells.

### Cell culture and treatment

The immortalized human chondrocyte cell line (IM-H488) was provided by Immocell Biotechnology Co., Ltd. (Xiamen, Fujian, China) and maintained in DMEM/F12 with 10% FBS (v/v) and 1% penicillin/streptomycin (v/v) as supplements. MIF Protein (Human, HY-P7387) was purchased from MCE (Shanghai, China). According to the experimental protocol, protein and total RNA were extracted after stimulating human immortalized cells with MIF for 24 h at a concentration of 50 ng/ml (30).

### Western blot analysis

Proteins were extracted using pre-cooled RIPA buffer (E121-01, GenStar, Shanghai, China) and quantified using the BCA Protein Assay Kit (PC0020, Solarbio, Beijing, China). Following separation via polyacrylamide gel, the proteins were transferred onto polyvinylidene difluoride (PVDF) membranes and then subjected to blocking with 5% bovine serum albumin (BSA, Sigma, USA). The anti-phospho-ERK1/2 (T40072), anti-ERK1/2 (T40071) and anti-PLA2G4A (MK62414) antibodies were obtained from Abmart (Shanghai, China). The primary antibodies were applied at a dilution of 1:2000 and incubated with the membranes overnight at 4°C. Following an overnight incubation, the membranes were then probed with the corresponding secondary antibody (1:5000 ratio) for at least 60 min at ambient temperature. Subsequently, visualization was accomplished by employing the ECL luminescence reagent (BL520A, Biosharp, Hefei, China). Quantification was done with Image Lab 6.0.

### Quantitative real-time PCR (qRT-PCR)

The human chondrocyte underwent lysis using of pre-cooled Trizol on ice for a duration of 5 min, followed by the extraction of their total RNA with chloroform. The RNA were then precipitated with isopropanol and resuspended in 75% pre-cooled ethanol. After dissolution in diethyl pyrocarbonate (DEPC)-treated water, RNA concentration was assessed using a NanoDrop Microvolume UV-Vis spectrophotometer (Thermo Fisher Scientific, Waltham, MA, USA). Reverse transcription was performed using the ReverTra ACE qRT-PCR RT Kit (TOYOBO, FSQ-101, Tokyo, Japan), and the resulting cDNA was quantified using Power Green qRT-PCR Mix (Dongsheng Biotech, P2102, Guangzhou, China) by 2^–ΔΔCt^ method. The primer sequences are given in Supplementary Table 3. Ultimately, 18S was selected as the internal control.

### Enzyme-linked immunosorbent assay (ELISA)

Cells were cultured in 6-well plates to approximately 80% confluence, and MIF was added at a final concentration of 50 ng/mL. After a 24-h incubation, supernatants were collected. The level of AA in the supernatants was immediately measured using the Human Arachidonic Acid (AA) ELISA Kit (CSB-E09040h, Cusabio Biotech, Wuhan, China).

### Statistical analysis

The experimental data are expressed as means ± standard error of the mean (SEM) from at least three independent experiments, and statistical analyses were performed using SPSS Statistics version 23. Differences between groups were assessed using one-way ANOVA, two-way ANOVA, and *t*-test, with a significance threshold set at *p* < 0.05.

## Results

### Chondrocyte gene expression reveals OA features

**FIGURE 1:**
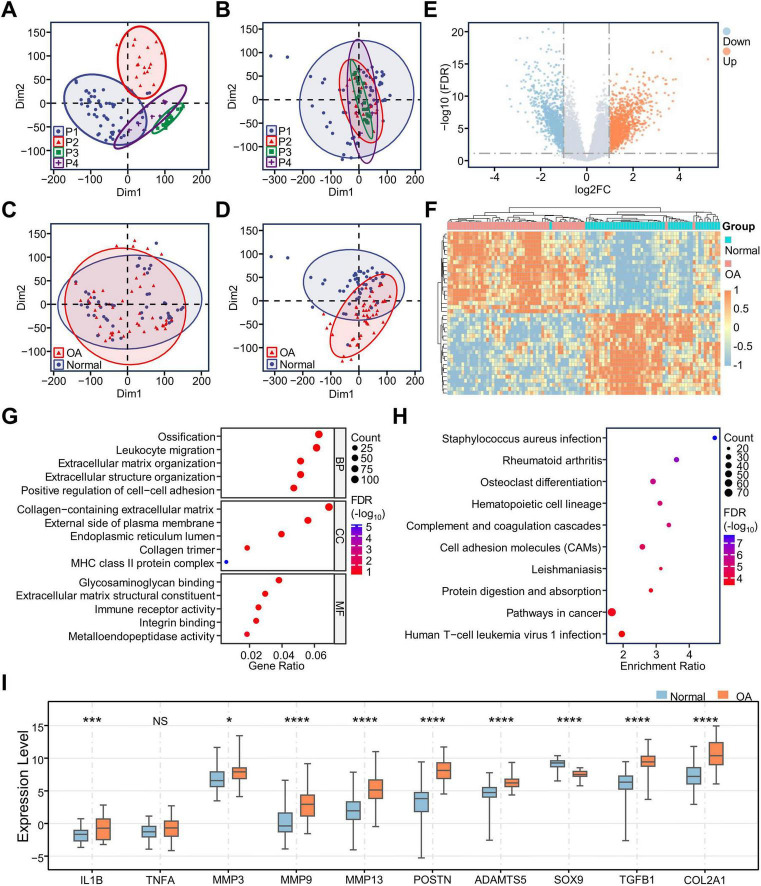
Integration of RNA-seq data and identification of DEGs. Scatter plots of the clustering of samples between different sequencing platforms before **(A)** and after **(B)** removing batch effects; Scatter plots of the sample clustering between OA and Normal groups before **(C)** and after **(D)** removing batch effects; **(E)** Volcano map showing differential expression of all genes; **(F)** Heatmap showing the expression of the 40 most significantly upregulated and downregulated genes in the OA samples; **(G)** GO enrichment analysis of DEGs demonstrating the results of BP/CC/MF analysis; **(H)** KEGG enrichment analysis of DEGs; **(I)** Box plots show the expression of important genes in OA cartilage. *p < 0.05; ***p < 0.001; ****p < 0.0001; NS, no significance; DEGs, differentially expressed genes; GO, gene ontology; KEGG, Kyoto Encyclopedia of Genes and Genomes; BP, Biological Process; CC, Cell Component; MF, Molecular Function.

We first integrated and normalized the count data matrices from the GSE114007, GSE168505, and E-MTAB-7313 datasets. Then, we found significant batch effects among the data from different platforms, and the group differences between the OA and normal groups were not significant. We used the removeBatchEffect function in the limma R package to effectively correct the batch effects. Meanwhile, significant group differences emerged between the OA and normal groups (Figures 1A–D). Next, we performed differential expression analysis on the corrected data using the limma R package. By setting the thresholds as | log2FC| > 1 and *p* < 0.05, we obtained 1,844 differentially expressed genes (DEGs), including 1,132 upregulated genes and 712 downregulated genes (Figure 1E). Then, we used a heatmap to show the expression of the top 40 DEGs. We found that the expression clustering was effective except for two OA samples and one Normal sample (Figure 1F).

To clarify the biological changes occurring in osteoarthritic chondrocytes, we conducted GO and KEGG analyses on all 1,844 DEGs. The results showed that ossification, leukocyte migration, extracellular matrix organization, positive regulation of cell-cell adhesion, staphylococcus aureus infection, rheumatoid arthritis, and osteoclast differentiation were enriched in osteoarthritic chondrocytes (Figures 1G, H), suggesting that chondrocytes may be involved in immune-regulatory activities and participate in extracellular matrix disintegration during OA. Moreover, we plotted boxplots using the data from the gene expression matrix after batch effects removal to observe the expression of classical genes in osteoarthritic chondrocytes (Figure 1I). The expression of IL1B, MMP3, MMP9, MMP13, ADAMTS5, and POSTN, associated with matrix catabolism, were elevated in OA. In genes associated with the promotion of synthetic metabolism, the expression of SOX9 is decreased in OA, while the expressions of TGFB1 and COL1A1 are increased in OA. These results were consistent with previous reports.

### Altered metabolism of chondrocyte in OA

**FIGURE 2:**
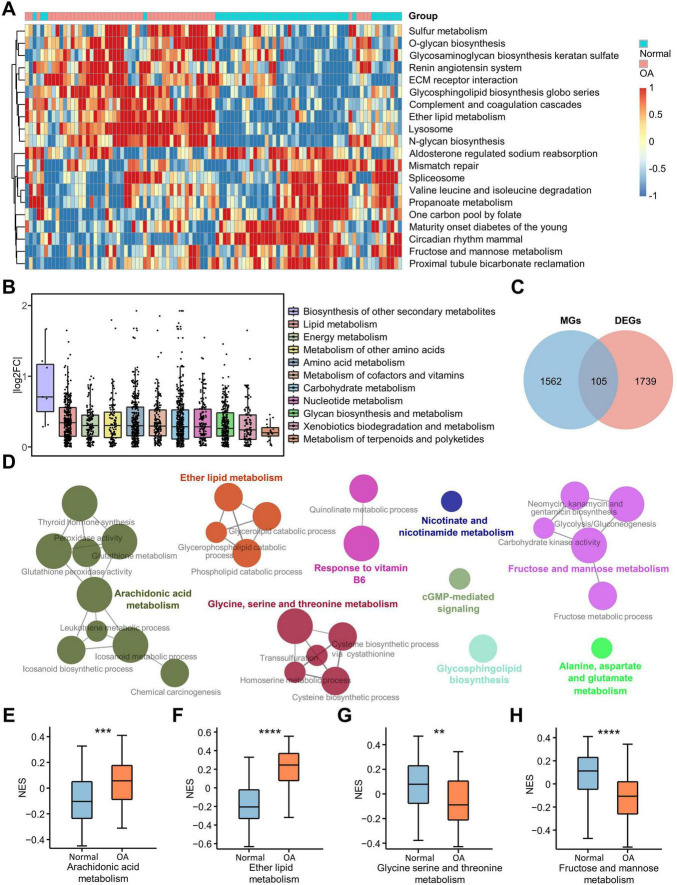
Differential expression of metabolism-related pathways. **(A)** Heatmap shows the 20 GSVA terms with the most pronounced up-regulation and down-regulation in OA; **(B)** Boxplot illustrates the differential expression of 11 human metabolism-related pathways downloaded from the KEGG database, with each point representing a gene in the pathway and the vertical coordinate representing the absolute value of the log2FC of the genes; **(C)** The Venn diagram shows the DEMGs obtained by taking the intersection of the DEGs and the metabolism-related genes; **(D)** Nine metabolic pathways were enriched for DEMGs in Cytoscape using ClueGO software; **(E–H)** The box plots show the enrichment scores of the four most significantly enriched metabolic pathways out of the nine major ones. **p < 0.01; ***p < 0.001; ****p < 0.0001. GSVA, gene set variation analysis; KEGG, Kyoto Encyclopedia of Genes and Genomes; DEGs, differentially expressed genes; DEMGs, differentially expressed metabolic genes.

To reveal the KEGG pathway differences between OA and normal chondrocytes, we performed GSVA analysis on all samples (Figure 2A). We found that eight of the twenty pathways with the most significant differences were related to metabolism. This suggests that metabolism plays an important role in the progression of OA. Therefore, to investigate the metabolic types affecting OA, we retrieved all the metabolic-related pathways for 11 metabolic types, including “Carbohydrate metabolism,” “Energy metabolism,” “Lipid metabolism,” “Nucleotide metabolism,” “Amino acid metabolism,” “Metabolism of other amino acids,” “Glycan biosynthesis and metabolism,” “Metabolism of cofactors and vitamins,” “Metabolism of terpenoids and polyketides,” “Biosynthesis of other secondary metabolites,” and “Xenobiotics biodegradation and metabolism,” from the KEGG pathway database (Supplementary File 1). Subsequently, we collected 83 metabolic pathways from the human KEGG signaling pathway using the KEGGREST package and identified 1,667 genes associated with these pathways (Supplementary File 2). Based on the value of log2FC between OA and normal groups calculated previously, we plotted boxplots for the log2FC of genes related to the 11 metabolic types. The results indicated that the “biosynthetic process of other secondary metabolites” showed the most significant difference, but only 7 genes were enriched in this process. In addition, “lipid metabolism” showed the most significant difference between OA and normal groups, followed by “Energy metabolism,” “Amino acid metabolism” and so on (Figure 2B). A Venn diagram was then constructed using DEGs and metabolism-related genes, resulting in 105 differentially expressed metabolic genes (DEMGs) (Figure 2C and Supplementary Table 4). The heatmap was used to visualize the expression of these genes (Supplementary Figure 1A) and the PPI network was constructed to show the interaction of these genes (Supplementary Figure 1B). Functional analysis of DEMGs conducted by Cytoscape plug-in ClueGo showed that nine cellular functions were enriched, including AA metabolism, ether lipid metabolism, glycine, serine and threonine metabolism, fructose and mannose metabolism, response to vitamin B6, nicotinate and nicotinamide metabolism, cGMP-mediated signaling, glycosphingolipid biosynthesis, and alanine, aspartate and glutamate metabolism (Figure 2D). Subsequently, we used boxplots to visualize the GSVA results of the four metabolic pathways and found that AA metabolism and ether lipid metabolism were activated in osteoarthritic chondrocytes, whereas glycine, serine and threonine metabolism and fructose and mannose metabolism were inhibited in osteoarthritic chondrocytes (Figures 2E–H). Furthermore, in OA, cGMP-mediated signaling and glycosphingolipid biosynthesis were activated, alanine, aspartate and glutamate metabolism was inhibited, and nicotinate and nicotinamide metabolism was not significantly changed (Supplementary Figures 1C–F). These results suggested that lipid metabolism, especially AA metabolism, plays an important role in OA.

### Metabolic pathways were enriched in OA-related gene module

**FIGURE 3:**
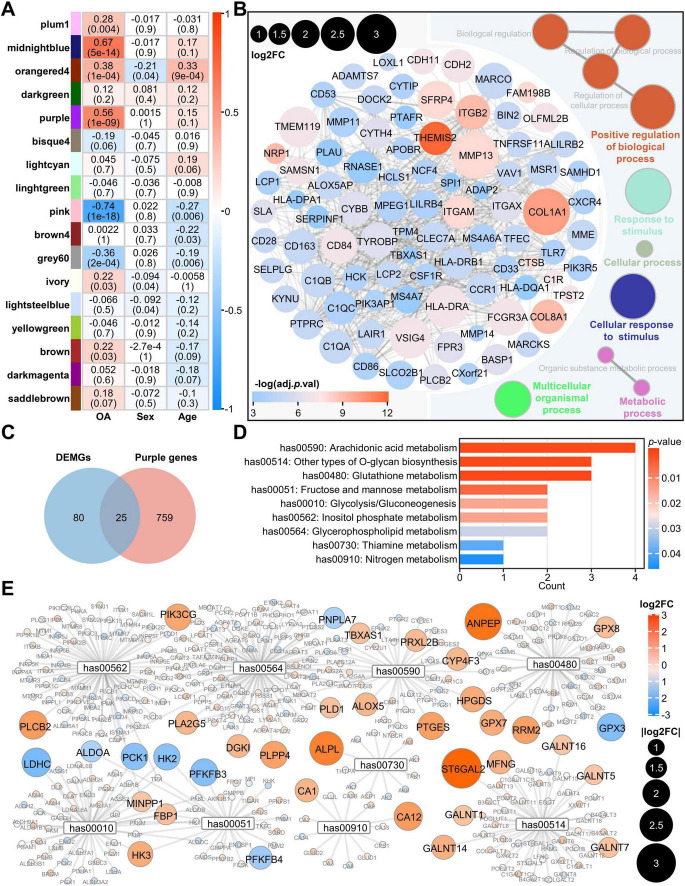
WGCNA analysis indicates enrichment of AA metabolism in OA cartilage. **(A)** Weighted gene co-expression network analysis. Rows represent gene modules. Columns represent sample traits. Each cell contains two values: a correlation coefficient between the module and sample trait and the associated *p*-value in parentheses. Significant correlations are color-coded according to the correlation coefficient, varying from high values in yellow to low values in blue; **(B)** PPI network of genes with log2FC greater than 1 in the purple module and functional visualization using ClueGO plugin; **(C)** The Venn plot shows 25 genes at the intersection of purple module genes and DEMGs; **(D)** KEGG analysis of the above 25 genes; **(E)** Network diagram of genes in the nine aforementioned pathways, in which the genes from the 105 DEMGs with log2FC greater than 1 are marked. The size of the circles is drawn from small to large according to their | log2FC| values, and the color ranges from blue (low value) to red (high value) based on the log2FC. PPI, protein-protein interaction; DEMGs, differentially expressed metabolic genes; KEGG, Kyoto Encyclopedia of Genes and Genomes.

To identify gene modules highly correlated with OA, we performed WGCNA to calculate the correlation between gene expression modules and sample traits. We found that the purple and the midnightblue modules were strongly positively correlated with OA, but not with age or sex (Figure 3A). Based on GS > 0.4 and MM > 0.8, we obtained 88 genes for the purple module and 23 genes for the midnightblue module as representative genes of respective modules (Supplementary Figures 2A, B). Subsequently, these representative genes were analyzed using STRING and ClueGO to investigate module-associated PPI network and functional enrichment. The results showed that the midnightblue module was mainly related to the process of cellular mitosis (Supplementary Figure 2C), whereas the purple module was associated with the cellular multi-biological processes, including metabolic processes (Figure 3B). According to previous findings that metabolic pathways were enriched in OA (Figure 2A), we chose the purple module to further investigate the relationship between metabolism and OA. We intersected the DEMGs with the purple module genes to obtain 25 OA-related metabolic genes (Figure 3C). Among these 25 genes, except for HS3ST3A1, the expression of the other genes was upregulated in OA cartilage (Supplementary Table 5). We performed a metabolic pathway enrichment analysis using the 25 genes and found that nine metabolic pathways were significantly enriched, including the AA metabolism pathway, other types of O-glycan biosynthesis, glutathione metabolism, fructose and mannose metabolism, glycolysis gluconeogenesis, inositol phosphate metabolism, glycerophospholipid metabolism, thiamine metabolism, and nitrogen metabolism (Figure 3D). Since AA metabolism is the most significant pathway among the nine enriched metabolic pathways related to the 25 genes, and consistent with previous studies (31), AA metabolism is closely associated with chondrocyte lesions in OA, the 25 genes may be closely associated with AA metabolism in OA cartilage. In addition, we extracted all the genes of these nine metabolic pathways and visualized their expression fold changes, and found that the expression of 37 genes was significantly altered, with more than 78% of them being upregulated in OA (Figure 3E). To further investigate the regulatory status of these pathways, we performed GSEA analysis. The results revealed that, except for Fructose and mannose metabolism, Glycolysis/gluconeogenesis, and Inositol phosphate metabolism, all other pathways were activated in OA (Supplementary Figure 2D).

### ProCs were the major cluster with enhanced AA metabolism

**FIGURE 4:**
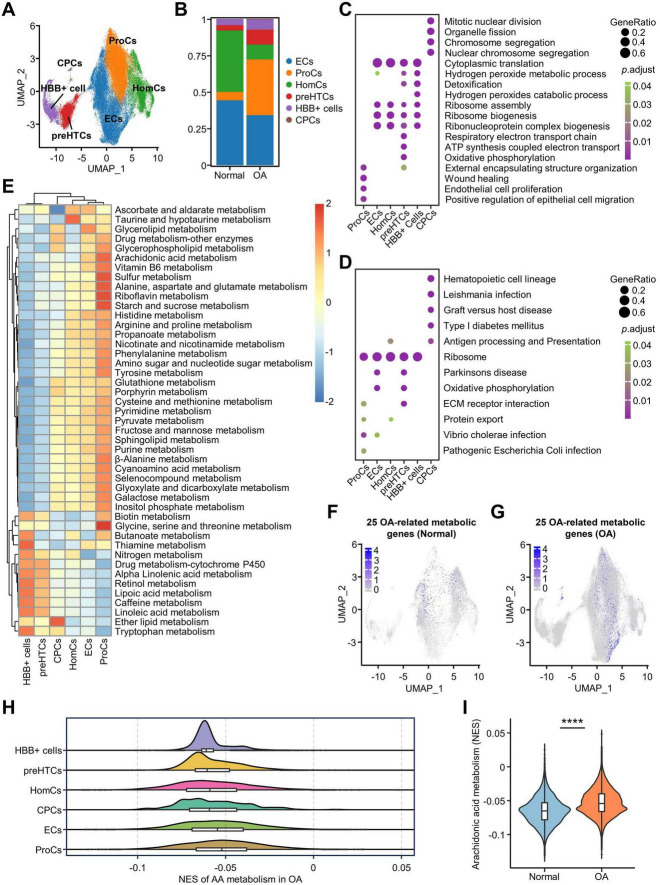
Single-cell analysis to clarify the metabolic characteristics of AA in different cell clusters. **(A)** Six cell clusters were annotated based on the UMAP algorithm and marker genes; **(B)** The bar plot shows the changes in the percentage of cell clusters in OA and Normal, with the *y*-axis representing percentages; **(C,D)** Bubble plots show the GO **(C)** and KEGG **(D)** analyses of DEGs in the clusters; **(E)** Heatmap showing differences in the expression of metabolic pathways in cell clusters analyzed by GSVA; **(F,G)** UMAP plot of the 25 genes in Normal and OA group; **(H)** The mountain range plot of differential expression of the 25 AA metabolism-related genes in cell clusters; **(I)** Differences in enrichment scores for AA metabolism in the Normal and OA groups in scRNA-seq data. ****p < 0.0001. OA, osteoarthritis; GO, gene ontology; KEGG, Kyoto Encyclopedia of Genes and Genomes; GSVA, gene set variation analysis; DEGs, differentially expressed genes; AA, arachidonic acid.

Having assessed the metabolic processes and associated genes in osteoarthritic chondrocytes, we used single-cell data to further investigate the changes in metabolism at the cellular resolution. We integrated scRNA-seq data using Harmony and showed that all samples were efficiently mapped (Supplementary Figure 3C). Through unsupervised clustering analysis, we presented a total of six cell subclusters numbered from 0 to 5 in the Uniform Manifold Approximation and Projection (UMAP) plot (Supplementary Figure 3A). Then, we used the FindAllMarkers function to obtain the characteristic genes of each cluster and the complete expression profiles of the marker genes (Supplementary File 3). We named these cell clusters with reference to the marker genes from multiple research findings (Figure 4A). Specifically, subcluster 1 was defined as ProCs and exhibits high expression of CLCF1, ASNS, and ARHGAP21 (11). Subcluster 2 was defined as HomCs, with high expression of FOSB, SNHG12, and DNAJB4 (11). Subcluster 5 was defined as CPCs, with high expression of CENPU, BIRC5, and STMN1 (11). Subcluster 3 was defined as preHTCs, which show high expression of MMP3 (32), COL2A1 (33), and ABI3BP (34). Subcluster 4 has high expression of HBB, HBA1, and HBA1. We defined it as HBB+ cells with reference to the study by Zhang et al. (35). Additionally, subcluster 0 (SQSTM1, PNO1, and RSL1D1) showed characteristics of highly metabolic cells (Supplementary Table 7), which is consistent with the function of ECs as defined by previous studies, thus we defined subcluster 0 as ECs (11, 36). We used a dot plot to concurrently show the cluster numbers before the definition, the assigned names post-definition, and the expression profiles of the corresponding markers (Supplementary Figure 3B). In addition, UMAPs of relevant markers have been utilized to support the demonstration (Supplementary Figure 4). The top 15 differentially expressed genes for each subcluster, including the marker genes, are listed in Supplementary Table 6. By comparing the changes in the proportions of each cell cluster in normal and osteoarthritic chondrocytes, we found that the proportions of the HBB+ cells, preHTCs, and ProCs cell clusters obviously increased in the OA group, whereas the proportions of HomCs and ECs decreased (Figure 4B). Furthermore, we performed GO and KEGG analysis based on the marker genes in each cell cluster of osteoarthritic chondrocytes to investigate the function of each cluster (Figures 4C, D). We found that the CPCs were associated with chromosome segregation, mitotic nuclear division, and nuclear chromosome segregation, indicating a differentiation potential. ECs and HomCs were involved in the regulation of the translation processes, such as ribosome assembly, ribonucleoprotein complex biogenesis, and ribosome biogenesis. ProCs and preHTCs were associated with the extracellular matrix (ECM) receptor interaction, whereas the hydrogen peroxide catabolism process was enriched in HBB+ cells.

In addition, we performed scGSVA analysis of metabolic pathways to characterize the regulation of metabolism in cell clusters and found that most metabolic processes were significantly enhanced in ProCs, including “AA metabolism,” “Vitamin B6 metabolism,” “Sulfur metabolism,” “Alanine, aspartate and glutamate metabolism,” “Riboflavin metabolism,” “Starch and sucrose metabolism,” and “Glycine, serine and threonine metabolism” (Figure 4E). To further investigate the expression patterns of the 105 DEMGs and 25 OA-related metabolic genes across different cell clusters, we utilized the addModuleScore function to integrate them into separate gene sets and visualized their distribution using UMAP plots. Specifically, the addModuleScore function calculates the average expression level of a given gene set at the single-cell level across different clusters, thereby reflecting the overall expression pattern of these genes. The results showed that the 25 OA-related metabolic genes are primarily expressed in ProCs of OA chondrocytes (Figures 4F, G and Supplementary Figure 3F). Similarly, the 105 DEMGs exhibit the same expression pattern (Supplementary Figures 3D, E, G). These findings suggest that metabolic regulation predominantly occurs in the ProCs. The mountain range plot showed that AA metabolism was activated in ProCs compared to the other clusters and the violin plot confirmed the significant differences in the AA metabolism pathway between normal and OA groups (Figures 4H, I), suggesting that the enhanced AA metabolism in OA group was associated with ProCs.

### Activation of the MIF pathway and increased expression of ERK and PLA2G4A were observed in ProCs

**FIGURE 5:**
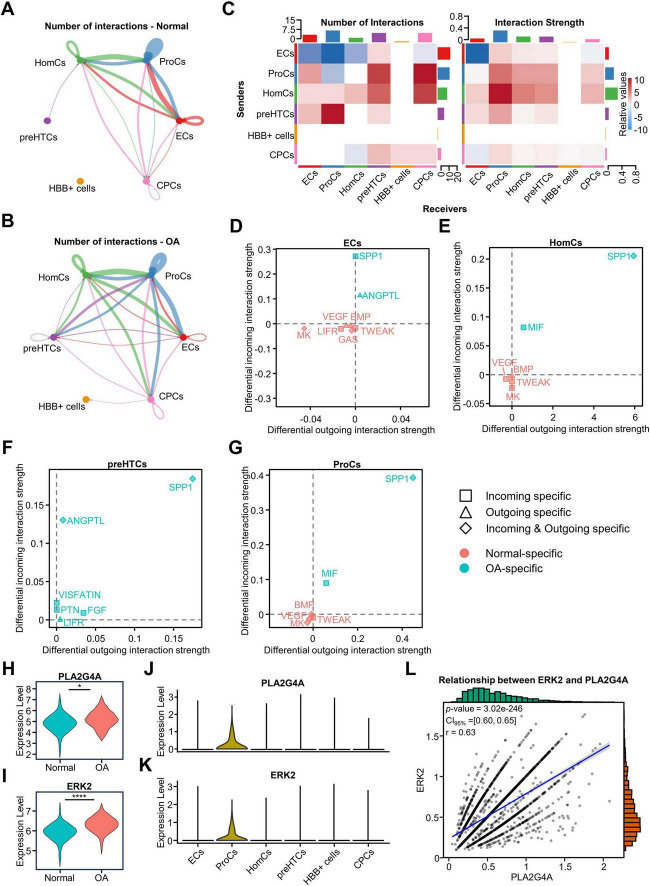
Intercellular communication reveals regulatory pathways of AA in ProCs in the OA group. **(A,B)** Interaction of communication between six cell clusters in the OA and Normal groups, the width of the line represents the strength of the interaction, and thicker lines indicate stronger signals. **(C)** Heatmap showing the number and weight of afferent and efferent signals for the six cellular subclusters, and the bar at the top and the side indicate the number and weight of the cells in the cluster as a receptor and a ligand in total. **(D–G)** Detailed information on the receptor and ligand pathways for each cluster. **(H,I)** Differential expression of PLA2G4A and ERK2 in Normal and OA in bulk RNA-seq data. **(J,K)** Expression of PLA2G4A and ERK2 in different cell clusters. **(L)** Correlation analysis of PLA2G4A and ERK2 expression in single-cell data. *p < 0.05; ****p < 0.0001. RNA-seq, RNA sequencing.

We investigated the changes in cellular communication among clusters of osteoarticular chondrocytes and their effect on metabolism using CellChat analysis. We calculated and visualized the cellular communication networks of the OA and normal groups, respectively, to investigate the strength and number of interactions between cells. The results of the circle plot showed that the interactions between clusters increased in the OA group, and the number of interactions in all clusters increased except ECs (Figures 5A, B). Therefore, we used a heatmap to show the changes of interaction in detail and found that ECs, as signaling senders, had diminished interactions with HomCs and ProCs, and HBB+ cells had almost no cellular communication with other clusters (Figure 5C). Furthermore, a scatter plot was used to visualize the dominant senders and receivers. We found that outgoing and incoming interaction strength of ProCs and HomCs were significantly increased in the OA group (Supplementary Figures 5A, B). Therefore, we further analyzed the changes of outgoing and incoming signals in all clusters for both the OA and normal groups, and found that the signaling between the OA and normal groups was significantly different in ECs, HomCs, preHTCs and ProCs, but not in CPCs, and HBB+ cells (Figures 5D–G). A previous study suggested that SPP1 signaling was one of the key factors in exacerbating the progression of OA (37). Consistent with this, the results showed that SPP1 signaling was detected in the four clusters in the OA group (Figures 5D–G). preHTCs, as a specific group that is in the late stage of OA, exacerbate the progression of OA, only expressed the OA-specific intercellular signals in our results (Figure 5F). In the OA group, we detected the secretion signals of angiopoietin-like protein (ANGPTL) ligands only in ECs and preHTCs, and the ANGPTL receptor signals were detected in preHTCs (Figures 5D, F). This indicates that both preHTCs and ECs are involved in the regulatory processes of lipid metabolism, extracellular matrix catabolism, and angiogenesis. Furthermore, we found that vascular endothelial growth factor (VEGF) signaling, bone morphogenetic protein (BMP) signaling, midkine (MK) signaling, and tumor necrosis factor–like weak inducer of apoptosis (TWEAK) signaling were significantly activated in ECs, ProCs, and HomCs of the normal group (Figures 5D–G). The coexistence of inhibitory and facilitative signals during the progression of OA suggests the presence of a dynamic equilibrium mechanism within osteoarthritic chondrocytes and indicates that there are extensive and complex interactions between the different chondrocyte clusters in OA patients.

Notably, we found that incoming MIF signaling was detected in HomCs and ProCs of the OA group (Figures 5E, G), suggesting that cells in this group received exogenous MIF stimuli and elicited relevant responses to a greater extent than other clusters. A previous study indicated that MIF activates the ERK signaling pathway to upregulate the expression of PLA2G4A, thereby catalyzing AA synthesis (10). Therefore, to investigate the relationship between MIF signaling and AA metabolism, we examined the expression of ERK2 and PLA2G4A in the bulk RNA-seq data. We found that the expression of ERK2 and PLA2G4A was higher in the OA group than in the normal group, and in ProCs than in other clusters, indicating that the involvement of MIF signaling in the regulation of AA metabolism may occur specifically in ProCs rather than in HomCs (Figures 5H–K). To determine whether ERK signaling pathway in ProCs was activated, we conducted a functional analysis using all the marker genes of ProCs. We selected the 10 terms with the highest number of enriched genes, and the results showed enrichment of “ERK1 and ERK2 cascade” and “regulation of ERK1 and ERK2 cascade” (Supplementary Figure 5C). This indicates that the ERK signaling pathway in ProCs is likely to be in an activated state. In addition, we used bulk RNA-seq data and scRNA-seq data to clarify the correlation between ERK2 and PLA2G4A, and found that they were highly correlated (Figure 5L and Supplementary Figure 5D). The results suggest that the MIF signaling-mediated ERK pathway may be involved in the increased metabolism of AA in ProCs.

### MIF activates the ERK/PLA2G4A pathway in chondrocytes and increases AA production

**FIGURE 6:**
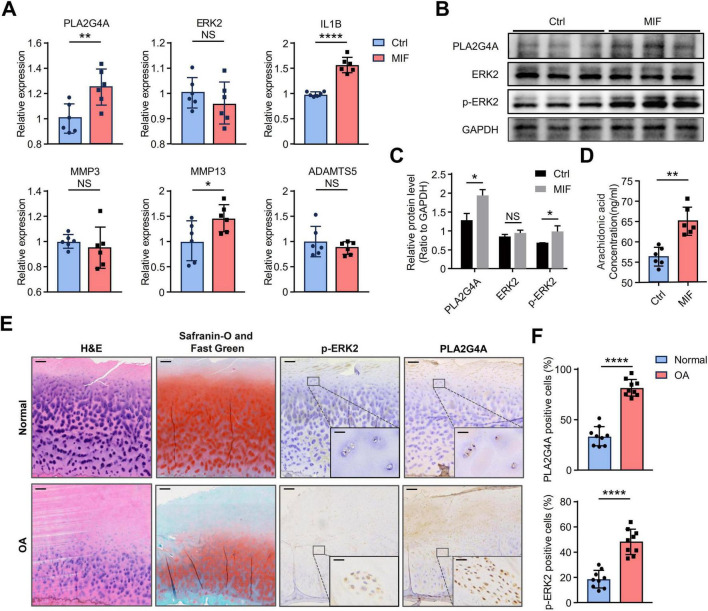
MIF stimulation activates the ERK/PLA2G4A pathway in chondrocytes, leading to increased AA production. **(A)** The mRNA levels of PLA2G4A, ERK2, IL1B, MMP3, MMP13, and ADAMTS5 in chondrocytes after 24 h of MIF treatment compared to control cells; **(B,C)** The relative protein expression levels of ERK2, p-ERK2 and PLA2G4A in cells after MIF stimulation were quantified by Western blotting; **(D)** AA level in the supernatant of chondrocytes treated with MIF compared to the control group; **(E)** H&E staining, Safranin-O and Fast Green staining and Immunohistochemical results in cartilages of p-ERK2 and PLA2G4A in Normal and OA groups. The direction of the joint cavity is at the top of the picture. Scale bars: 200 μm for the whole image and 25 μm for the enlarged area; **(F)** Bar chart presenting a comparative quantitative analysis of IHC-positively stained cells between OA group and Normal group. NA, no significance; *p < 0.05; **p < 0.01; ****p < 0.0001. AA, arachidonic acid.

To validate our analytical results, we treated human immortalized chondrocytes with MIF protein and detected the changes in the mRNA levels of PLA2G4A and ERK2. The results showed that MIF stimulation led to an up-regulation of PLA2G4A expression in chondrocytes, while the expression level of ERK2 did not increase (Figure 6A). Since phosphorylated ERK2 represents its activated state, we next verified whether the phosphorylation level of ERK2 had changed. We detected the protein levels of PLA2G4A, ERK2, and p-ERK2 after MIF stimulation of chondrocytes. The results showed that the protein levels of PLA2G4A and p-ERK2 increased, while the protein level of ERK2 remained unchanged (Figures 6B, C). These results indicate that MIF activates the ERK signaling pathway by mediating the phosphorylation of ERK2. Next, to investigate whether MIF promotes the production of AA in chondrocytes, we evaluated the changes in AA content in human immortalized chondrocytes after MIF stimulation. The results showed that the production of AA increased significantly in MIF-stimulated chondrocytes (Figure 6D). In addition, we found that MIF treatment led to an increase in the expression of IL1B and MMP13 genes (Figure 6A), suggesting that MIF may play a promoting role in the progression of OA.

We next attempted to clarify whether the ERK/PLA2G4A signaling pathway is activated in OA cartilage through IHC. We obtained knee joint cartilage samples from OA patients who underwent total knee arthroplasty and divided them into two groups: the OA group and the Normal group, with three samples in each group. For each observation index, we randomly selected three high-power fields (magnification × 20) from each sample, and a total of 9 high-power fields were used for observation and statistical analysis (Supplementary Figure 6). H&E staining revealed that, compared to the normal group, the OA group exhibited a decreased total number of chondrocytes, reduced cartilage thickness, a significantly increased number of cells in the fibrous and proliferative layers, a disordered cartilage matrix network, and damaged cartilage structure. Meanwhile, Safranin-O and Fast Green staining showed that the content of sulfated proteins and dextran decreased in the OA group, and cartilage ossification was present (Figure 6E). These results indicate that cartilage degeneration is severe in the OA group. The results of immunohistochemical staining showed that the protein levels of PLA2G4A and p-ERK2 in the OA group were significantly higher than those in the Normal group (Figures 6E, F). This indicates that the ERK/PLA2G4A signaling pathway is significantly activated in OA cartilage.

## Discussion

Previous studies have shown that metabolites of AA, such as prostaglandin E2 (PGE2) and leukotrienes, play a pro-inflammatory role in the progression of OA. Almost all joint tissues such as synovium, cartilage, meniscus, and subchondral bone are involved in the release of these inflammatory mediators (8, 38). Furthermore, osteoarthritic chondrocytes have been classified into several groups by single-cell analysis studies (11). However, the metabolism of AA and its interaction mechanism in different clusters of osteoarthritic chondrocytes have never been reported. In our study, we found that MIF-mediated activation of ERK/PLA2G4A could cause an increase in AA production in chondrocytes through our analysis and experimental validation. This may be an important factor in the transformation of ProCs into unfavorable cells while also driving the progression of OA. In addition, MIF induced increased expression of IL1B and MMP13, which can induce inflammatory responses and degradation of cartilage matrix. Our results suggest that MIF may drive OA progression and may be a therapeutic or preventive target for OA.

AA is the primary n-6 PUFA found in inflammatory cells, leading to the production of inflammatory eicosanoids (6). Attur et al. (39) in their metabolite analysis of supernatants from normal and osteoarthritic chondrocytes concluded that increased levels of PGE2 and LTB4 are produced in OA cartilage compared to normal cartilage. Here, we integrated and analyzed bulk RNA-seq data and scRNA-seq data to show that AA metabolism is more significantly enriched in metabolism-related pathways in chondrocytes compared to other pathways. This confirms the important role of increased AA metabolism in OA chondrocyte metabolism. PLA2G4A is an upstream regulator of the eicosanoid pathway that can release free AA from the sn-2 position of membrane phospholipids (40). Numerous studies have confirmed that MIF can activate the ERK/PLA2G4A pathway to induce increased AA production. In rheumatoid arthritis (RA) fibroblastic-like synoviocytes (FLS), MIF upregulates PLA2G4A activity and PLA2G4A mRNA expression (41). In the NIH/3T3 fibroblast cell line, MIF promotes the sustained activation of ERK pathway, which is associated with the activation of PLA2G4A (30). In our analysis, we identified activation of the MIF pathway and increased expression of ERK2 and PLA2G4A, suggesting the possibility that this pathway may be activated in chondrocytes. Subsequently, the increased levels of ERK2 phosphorylation and PLA2G4A expression were confirmed by WB experiments after MIF stimulation of chondrocytes, and an increase in AA concentration was determined by Elisa. Taken together, we can conclude that the MIF-mediated ERK/PLA2G4A pathway is involved in the increased AA production in osteoarthritic chondrocytes. Surprisingly, our qPCR results showed that MIF stimulation did not induce changes in ERK2 expression in chondrocytes, which is inconsistent with our previous analysis. We speculate that this might be because the relatively complex pathophysiological environment in osteoarthritic cartilage leads to the increased expression of ERK2, while MIF is mainly involved in regulating its phosphorylation process.

In our scRNA-seq data analysis, we classified chondrocytes into six clusters, which differ from the findings of Ji et al. (11). First, we did not separate the mast cell cluster (HTC). Our explanation is that these two clusters have similar cellular properties enriched for processes such as lipid metabolism, vascular endothelial production, and inflammatory cell migration, thus allowing the two to produce blurred cluster boundaries. In addition, we isolated the CPCs and, together with the results of our functional analysis, considered them to be a cell lineage with the potential to divide and differentiate. This difference in cell clustering illustrates that there is no unified standard for the classification of chondrocyte sub-clusters and no classical markers for cell clustering. Even when other scholars reference the clustering method of Ji et al. (11), it is difficult to achieve consistent clustering results. Moreover, some new cell types have been further identified (42, 43). Despite these uncertainties, in combination with the functional characteristics of the cell clusters, we can still infer that the three cell clusters, ProCs, preHTCs, and HBB+ cells, are closely associated with pro-inflammatory and cartilage matrix catabolism.

CellChat analysis can predict cell-to-cell communication pathways. It uses a database with info on ligand-receptor complexes and regulators. By combining math models, gene expression analysis, and stats, it reveals how cells communicate with the external microenvironment at the single-cell level. Through CellChat analysis, we identified incoming MIF signaling in ProCs from the OA group. This indicates that ProCs are capable of receiving external MIF signals, thereby triggering changes in certain intracellular signaling pathways. In our cell experiments, we found that MIF stimulation could activate the ERK/PLA2G4A pathway in chondrocytes, leading to an increase in AA production. Therefore, we speculate that ProCs may be the main chondrocytes that receive MIF stimulation in OA. Ji et al. (11, 44) found that ProCs are characterized by being flat and columnar, and are mainly distributed within the proliferative zone, which is located in the middle area of the articular cartilage. In OA cartilage, due to joint wear, ProCs are closer to the joint cavity, which allows MIF in the synovial fluid to directly act on ProCs through physical infiltration. This may be one of the key factors in the formation of ProCs during the progression of OA. In turn, the increased production of AA in ProCs will also act as a chronic toxin and further promote the progression of OA. However, this hypothesis needs to be further validated in future studies.

MIF occupies an apex position in the regulation of the immune response and is involved in numerous immune-related processes (45). However, research into the role of MIF in OA has been inconsistent, with some studies reporting conflicting results. A study of blood samples from 119 end-stage knee/hip OA patients found that MIF may play a protective role in OA. In contrast, Rowe et al. (46, 47) suggested that inhibition of MIF reduces the severity of age-related OA. In this study, we demonstrate that MIF can activate the ERK/PLA2G4A pathway to induce increased AA production. In addition, in our experiments we found that MIF stimulation of chondrocytes increased the expression of MMP13 and IL1B. IL1B can trigger an inflammatory cascade response that induces chondrocytes to produce collagen-degrading enzymes such as MMP3, MMP9, MMP13 and the proteoglycan-degrading enzyme ADAMTS5 (48, 49). The expression of these genes is involved in the degradation of the cartilage matrix in OA.

However, our study has several limitations. For example, the precise cellular clustering of both osteoarthritic cartilage samples and osteoarthritic chondrocyte models, as well as the precise identification of ProCs, require further investigation.

## Conclusion

In conclusion, we found that MIF can increase the production of IL1B and MMP13 in chondrocytes, thereby promoting the progression of OA. Additionally, MIF can also enhance AA production by activating the ERK/PLA2G4A signaling pathway in OA, which may be closely associated with ProCs in osteoarthritic cartilage. Therefore, targeted inhibition of MIF signal transduction in chondrocytes, especially in ProCs, may represent a novel strategy for the prevention and treatment of OA.

## Data Availability

Publicly available datasets were analyzed in this study. This data can be found here: The datasets presented in this study can be found in online repositories (https://www.ncbi.nlm.nih.gov/geo/; https://www.ebi.ac.uk/).

## Footnotes

1
https://www.ncbi.nlm.nih.gov

2
https://www.ebi.ac.uk/

3
http://www.kegg.jp/kegg/
