# Supplementary material for: Integrative analysis of bulk and single-cell RNA sequencing data reveals increased arachidonic acid metabolism in osteoarthritic chondrocytes
Source: Front Med (Lausanne). 2025 May 9;12:1552029. doi: 10.3389/fmed.2025.1552029 (PMC12098391; doi:10.3389/fmed.2025.1552029)
Supplement: Supplementary file 4 [file Data_Sheet_4.docx]

Supplementary Materials

Integrative Analysis of Bulk and Single-cell RNA Sequencing Data Reveals Increased Arachidonic Acid Metabolism in Osteoarthritic Chondrocytes

Kan Wu1, Zhaoqian Zhong, Li Chen, Haihua Luo, Aolin Jiang, Linlin Tao, Yong Jiang*

*Correspondence: Yong Jiang: [jiang48231@163.com](mailto:jiang48231@163.com)

**This file includes**:

Supplementary Figure 1-6

Supplementary Tables: Table S1-S7

## Supplementary Figures

**
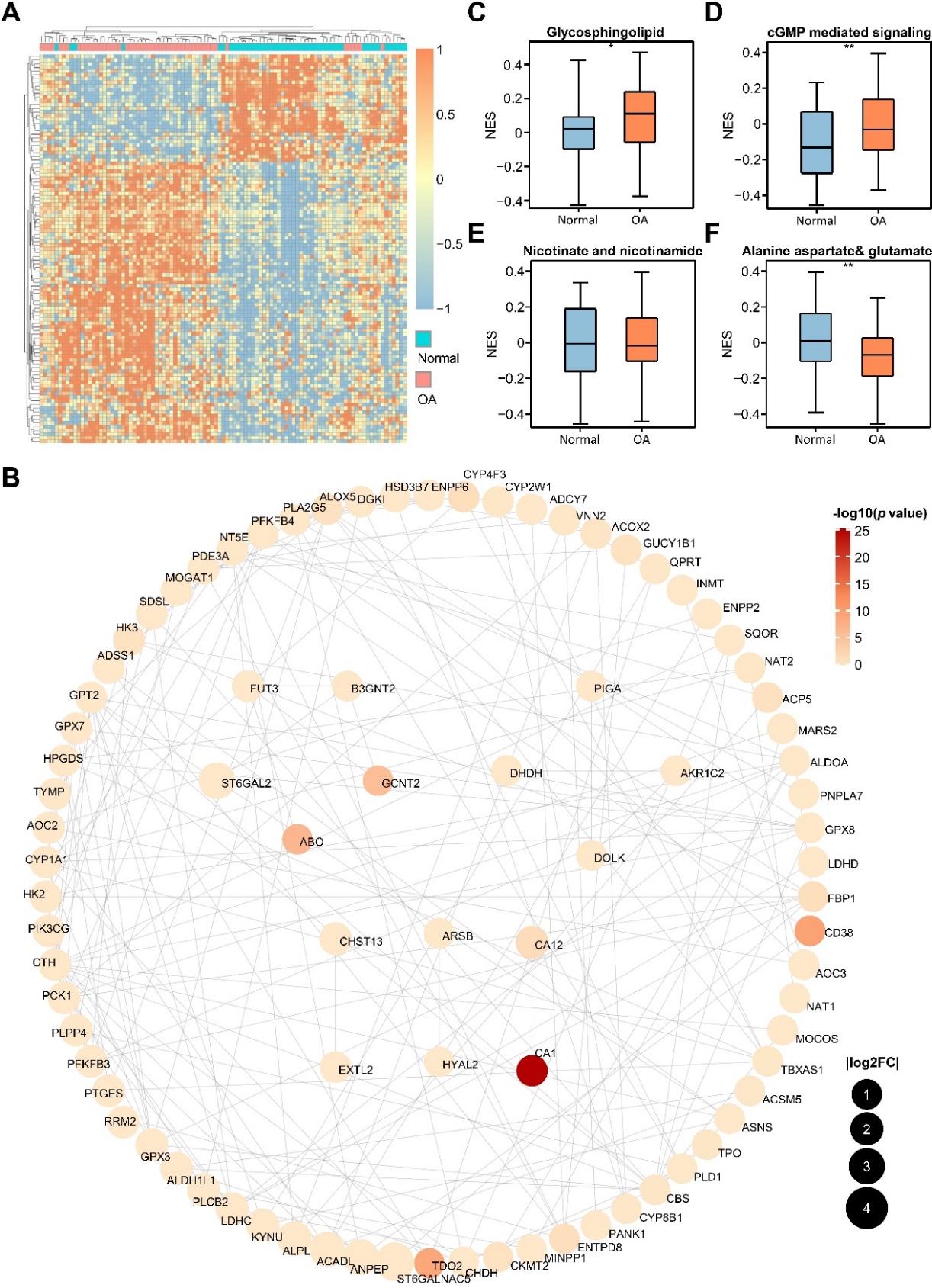
**

**Supplementary Figure 1. Differential expression of DEMGs and PPI network. (A)** Heatmap of DEMGs showing their differential profiles in OA and Normal groups; **(B)** The PPI network of the 105 DEMGs; **(C-F)** The inhibitory and activation statuses of the other four metabolic pathways in osteoarthritis (OA) and normal conditions. *, *p* < 0.05; **, *p* < 0.01.


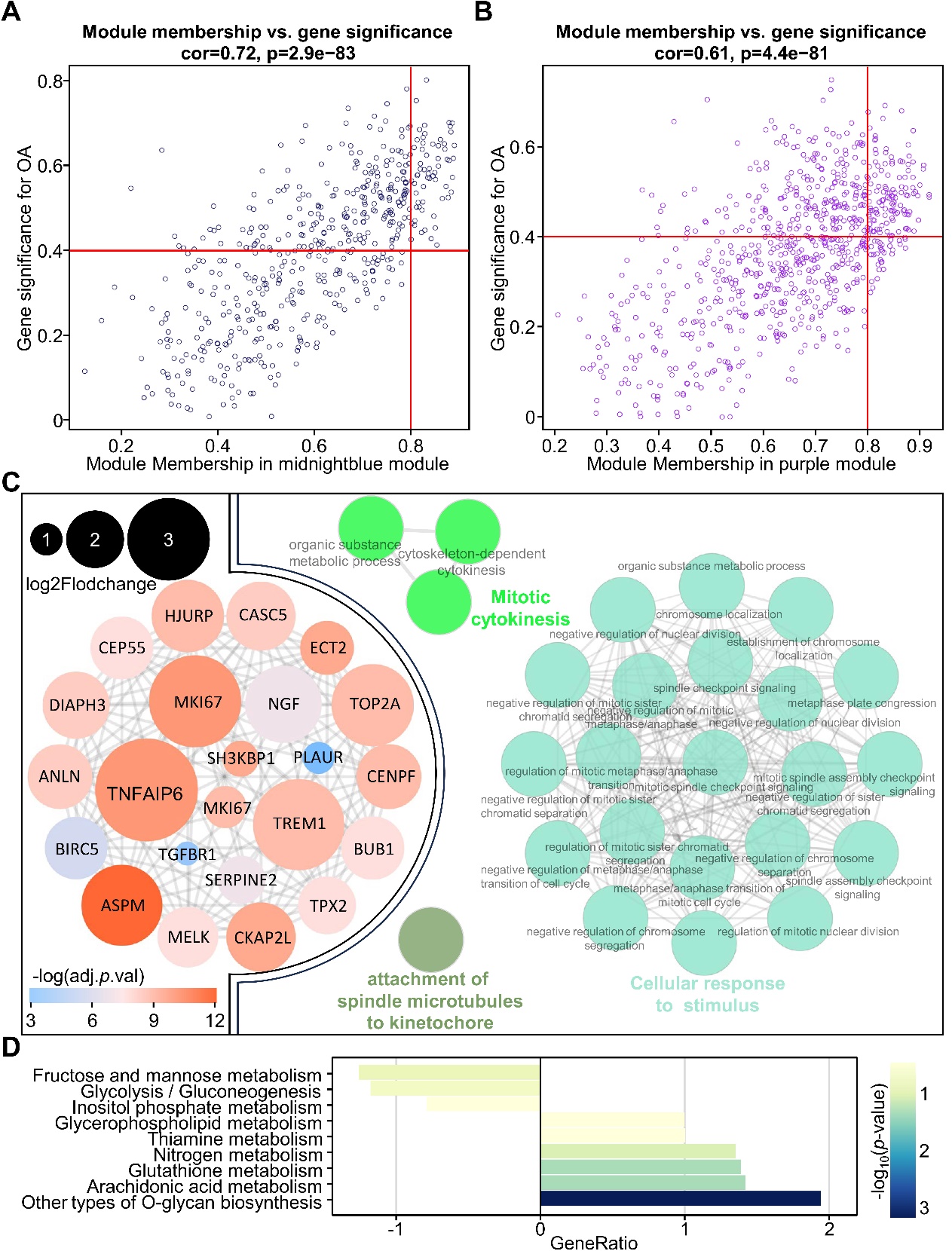


**Supplementary Figure 2. WGCNA screening key modules. (A)** Gene screening conditions in the midnightblue module; **(B)** Gene screening conditions in the purple module; **(C)** PPI network and functional analysis of the genes screened in the Midnightblue module; **(D)** GSEA analysis revealed the upregulation and downregulation status of the nine metabolic pathways.

**
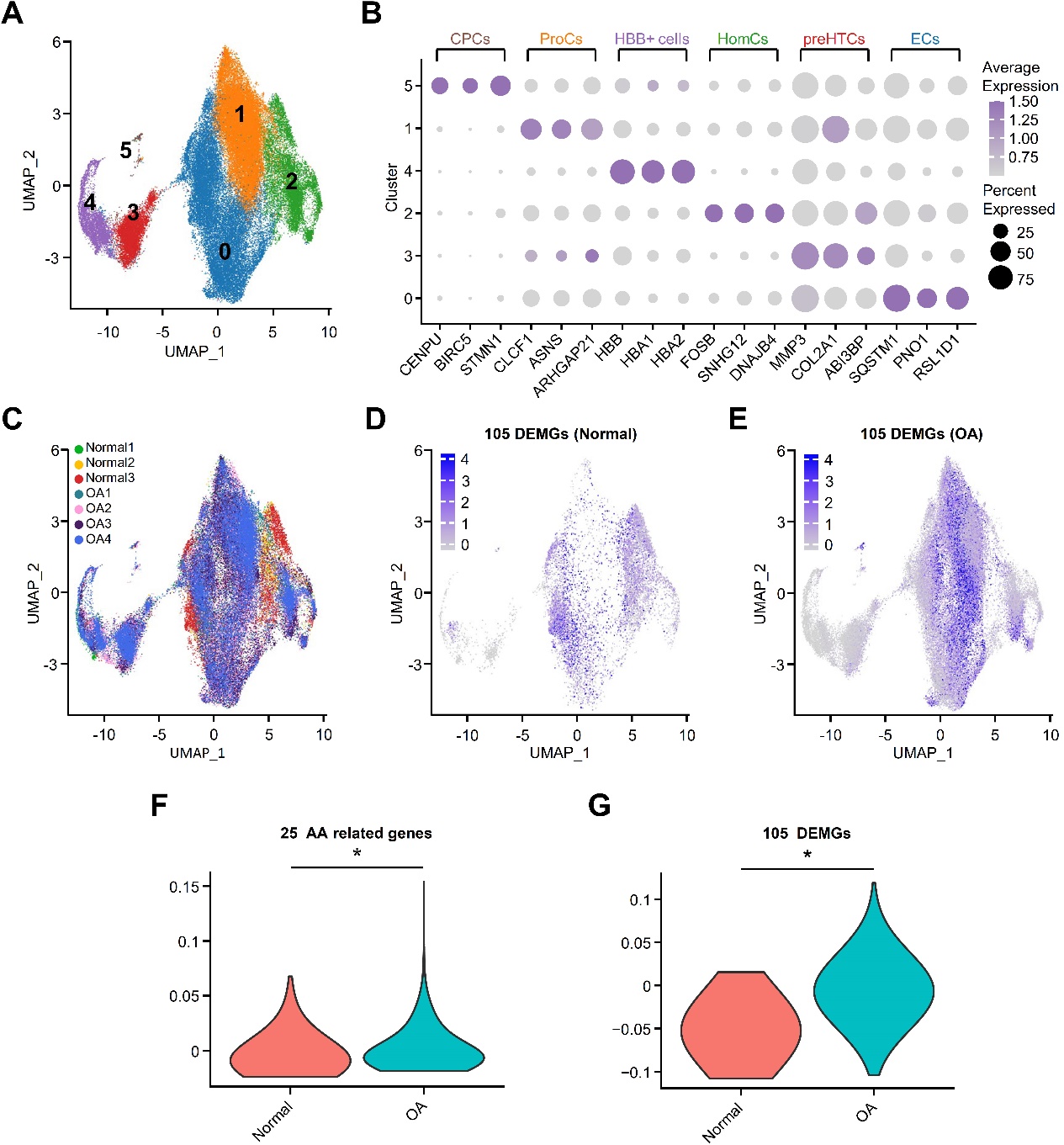
**

**Supplementary Figure 3. Marker genes for cell clusters and UMAP of genes. (A)** UMAP plot showing the chondrocytes divided into subclusters 0-5; **(B)** Dot plot show the cluster numbers before the definition, the assigned names post-definition, and the expression profiles of the corresponding markers; **(C)** UMAP projection of each sample in scRNA-seq data; **(D, E)** UMAP projections of the 105 DEMGs in Normal and OA samples; **(F, G)** Violin plots comparing the expression of 25 OA-related metabolic genes and 105 DEMGs in ProCs in scRNA-seq data.


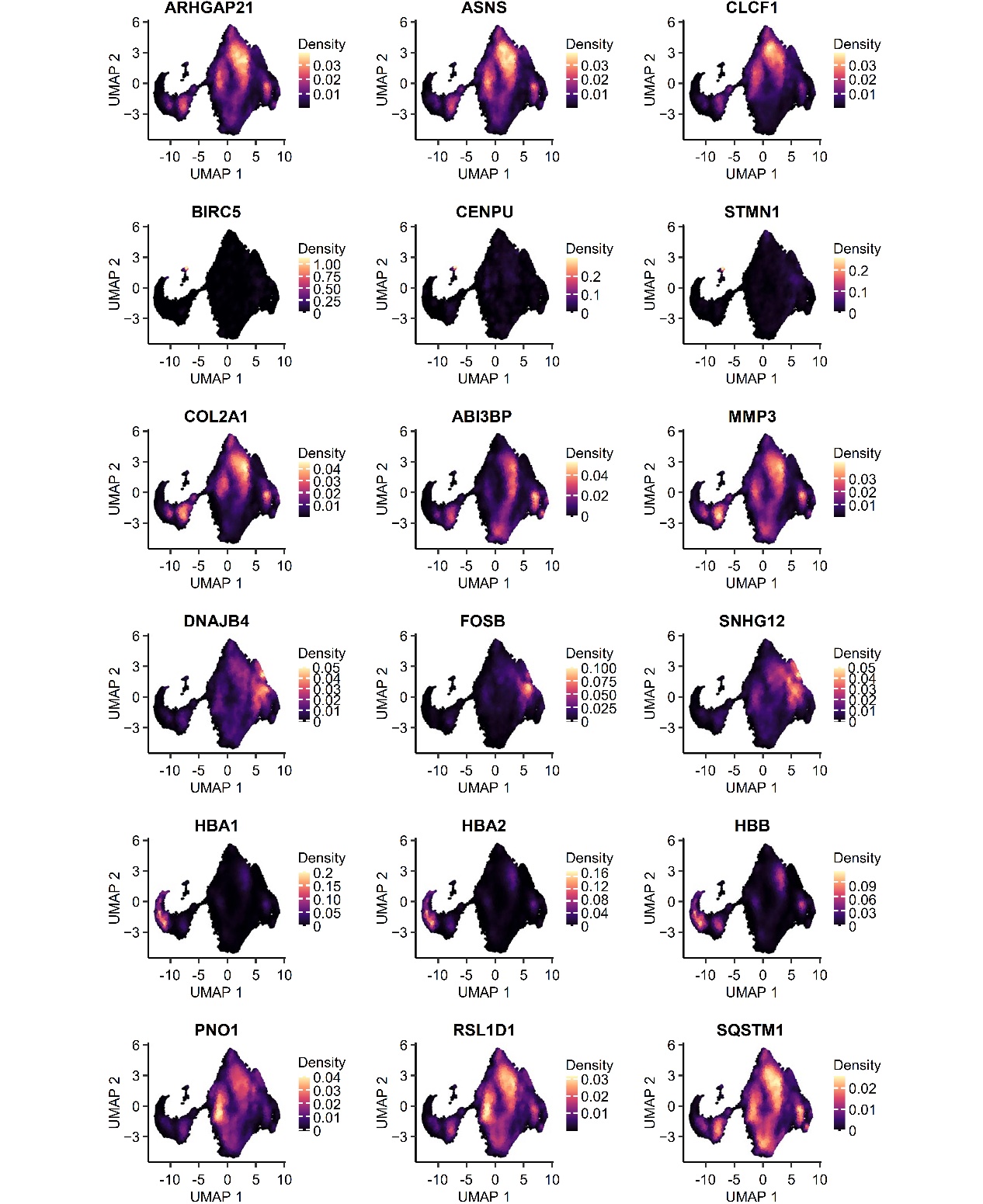


**Supplementary Figure 4. Distribution of cell markers at specific locations on UMAP plot.** Three marker genes are presented for each cluster. From top to bottom, they are the marker genes of ProCs, CPCs, preHTCs, HomCs, HBB+ cells, and ECs respectively.


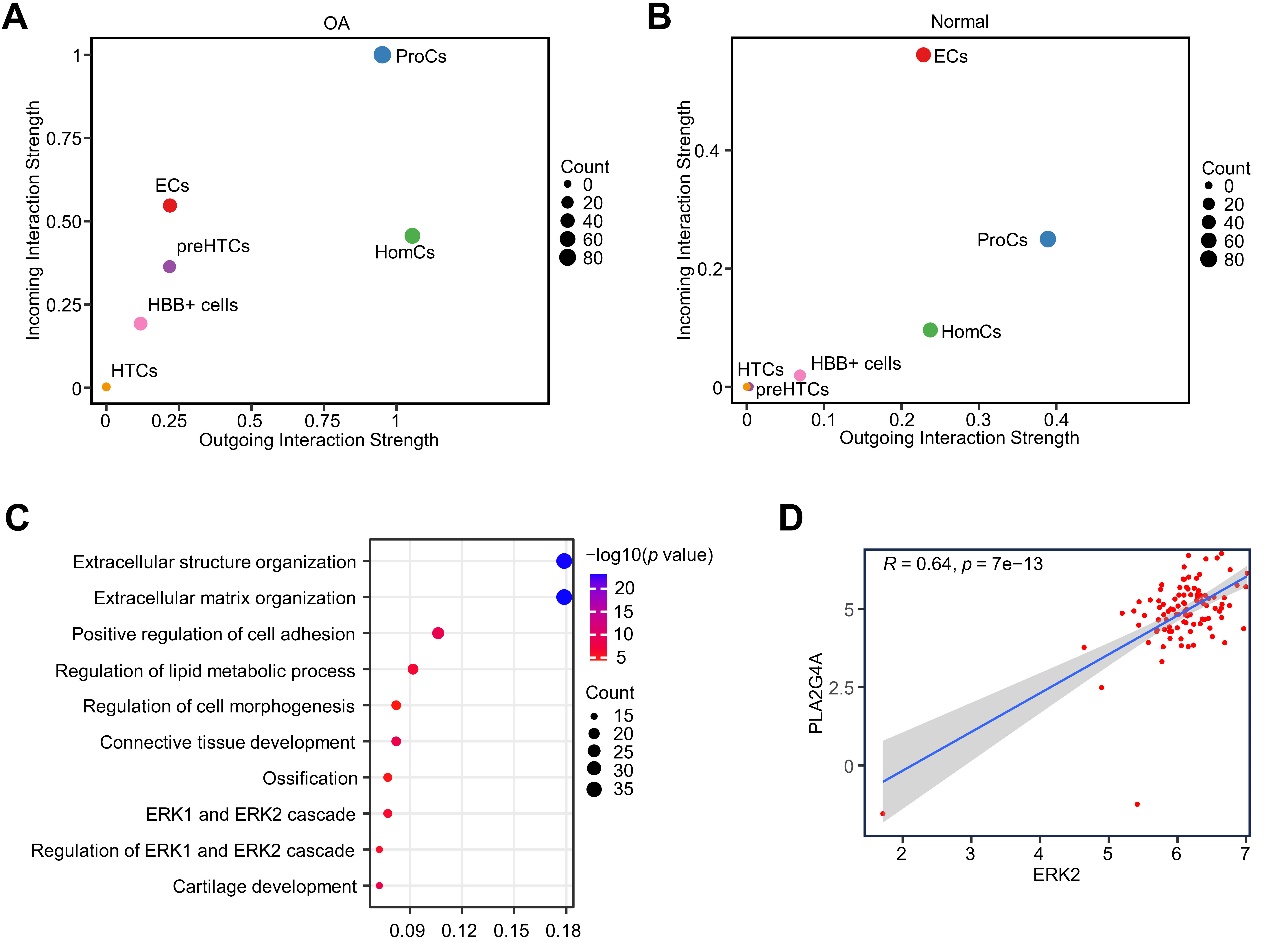


**Supplementary Figure 5. CellChat analysis reveals pathways of increased AA metabolism.**

**(A, B)** Dot plots illustrating the strength of cell clusters as receptors or ligands in intercellular communication processes; **(C)** Results of GO enrichment analysis of marker genes for ProCs; **(D)** Results of correlation analysis of PLA2G4A and ERK2 in the RNA-seq.


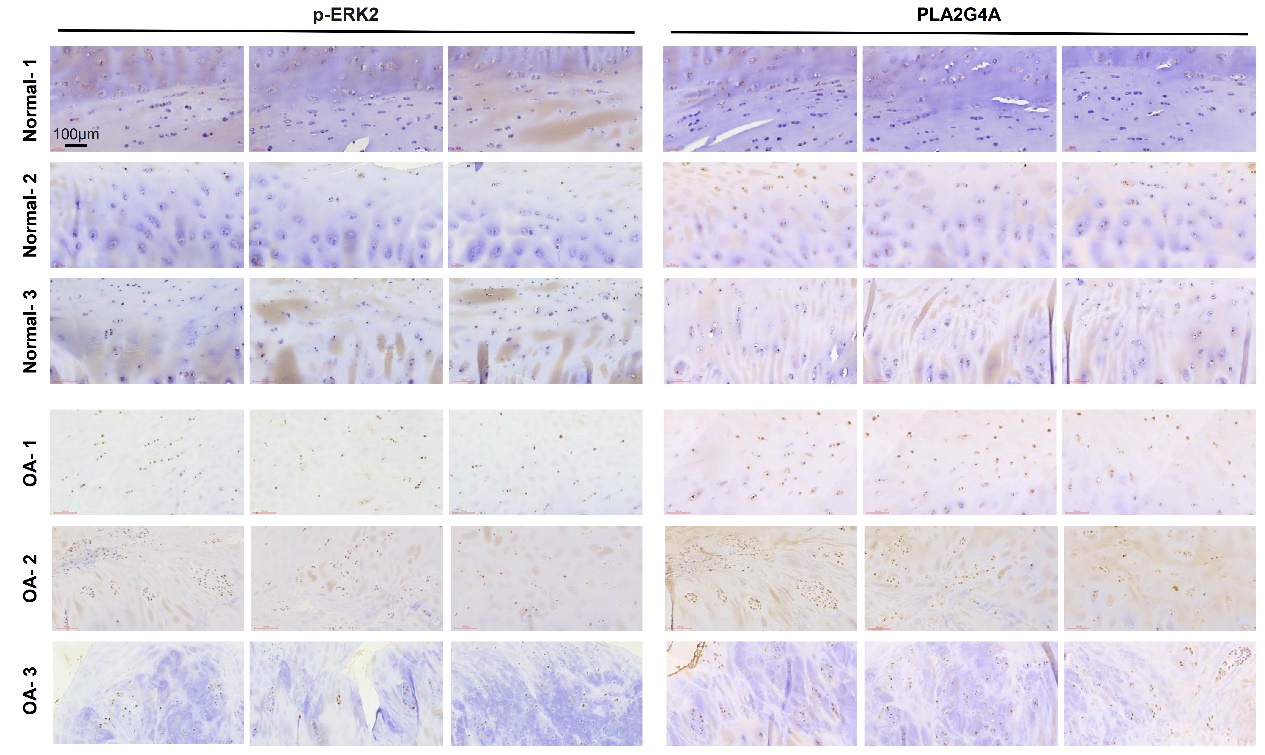


**Supplementary Figure 6. IHC staining of different areas in OA cartilage and Normal cartilage.**

Normal-1, Normal-2, and Normal-3 are three samples of normal cartilage. OA-1, OA-2, OA-3 are three samples of OA cartilage. Three high magnification fields of view were randomly selected for each assay in each sample.

**Supplementary Tables**

| **Table S1** **Information of public data** | | | | |
| --- | --- | --- | --- | --- |
| **ID** | **Platform** | **Participants** | **Tissues** | **Data type** |
| GSE114007 | Illumina HiSeq 2000  Illumina NextSeq 500 | 10 OA and 8 Normal  10 OA and 10 Normal | Cartilage | bulk RNA-Seq |
|  |  |  | Cartilage | bulk RNA-Seq |
| GSE168505 | Illumina HiSeq 2500 | 4 OA and 3 Normal | Cartilage | bulk RNA-Seq |
| E-MTAB-7313 | Illumina HiSeq 4000 | 27 OA and 27 Normal | Cartilage | bulk RNA-Seq |
| GSE169454 | Illumina HiSeq 2500 | 4 OA and 3 Normal | Cartilage | scRNA-Seq |

| **Table S2** **Information of patients and samples** | | |  | |
| --- | --- | --- | --- | --- |
| **Patients** | **Group** | **Age (years** **old)** | | **Tissues** |
| Patient 1 | Normal | 65 | | Knee |
|  | OA |  |  | Knee |
| Patient 2 | Normal | 60 | | Knee |
|  | OA |  |  | Knee |
| Patient 3 | Normal | 62 | | Knee |
|  | OA |  |  | Knee |

| **Table S3 Primer sequences of qRT-PCR** | | | |
| --- | --- | --- | --- |
| **Genes** | **Primer** |  | **Sequence** |
| 18s | Forward  Reverse |  | AGTCCCTGCCCTTTGTACACA  CGATCCGAGGGCCTCACTA |
| IL1B | Forward  Reverse |  | CCACAGACCTTCCAGGAGAATG  GTGCAGTTCAGTGATCGTACAGG |
| MMP3 | Forward  Reverse |  | CACTCACAGACCTGACTCGGTT  AAGCAGGATCACAGTTGGCTGG |
| MMP13 | Forward  Reverse |  | CCTTGATGCCATTACCAGTCTCC  AAACAGCTCCGCATCAACCTGC |
| ADAMTS5 | Forward  Reverse |  | CCTGGTCCAAATGCACTTCAGC  TCGTAGGTCTGTCCTGGGAGTT |
| ERK2 | Forward  Reverse |  | ACACCAACCTCTCGTACATCGG  TGGCAGTAGGTCTGGTGCTCAA |
| PLA2G4A | Forward  Reverse |  | GGATTCTCTGGTGTGATGAAGGC  CCTTTCTCTGGAAAATCAGGGTG |

| **Table S4** **Differential expression of 105 DEMGs in bulk RNA-seq data from OA cartilage** | | | |
| --- | --- | --- | --- |
| **Genes** | **logFC** | ***p*.Value** | **adj.*p*.Val** |
| HK3 | 1.541972476 | 0.0000707 | 0.000438437 |
| HK2 | -1.668581949 | 1.89E-25 | 4.33E-21 |
| FBP1 | 1.13489171 | 0.000147679 | 0.000813924 |
| ALDOA | -1.147998313 | 6.43E-09 | 0.000000166 |
| LDHC | -1.924354066 | 9.68E-11 | 4.72E-09 |
| MINPP1 | 1.023443033 | 0.00000122 | 0.0000138 |
| PCK1 | -1.702385307 | 0.000000367 | 0.00000507 |
| DCXR | -1.004766554 | 3.31E-09 | 9.36E-08 |
| DHDH | -1.245811548 | 0.00000239 | 0.0000246 |
| PFKFB3 | -1.75148576 | 3.65E-21 | 6.42E-18 |
| PFKFB4 | -1.340653616 | 2.58E-12 | 2.28E-10 |
| ACADL | -2.554307927 | 5.82E-14 | 9.78E-12 |
| LIPA | 1.785267726 | 3.85E-13 | 4.63E-11 |
| HSD3B7 | 1.304799455 | 0.00000125 | 0.0000141 |
| CYP8B1 | 1.052602227 | 0.0000153 | 0.000118768 |
| ACOX2 | 1.251850236 | 0.0000489 | 0.000319602 |
| AKR1C2 | 1.126145613 | 2.14E-08 | 0.000000458 |
| HSD11B2 | -1.018779102 | 0.000258487 | 0.001306546 |
| CYP1A1 | -1.647584673 | 2.75E-10 | 1.13E-08 |
| GPT2 | -1.550917029 | 1.5E-21 | 3.81E-18 |
| NT5E | 1.420545396 | 1.81E-13 | 2.54E-11 |
| ENTPD8 | -1.037395587 | 0.000135672 | 0.000758463 |
| RRM2 | 1.764320106 | 0.00000001 | 0.000000241 |
| ADCY7 | 1.259723431 | 0.00000157 | 0.0000171 |
| GUCY1B1 | 1.248038732 | 0.0000762 | 0.000468098 |
| PDE3A | 1.435261491 | 0.00000114 | 0.0000131 |
| ADSS1 | -1.546630043 | 4.97E-14 | 8.74E-12 |
| NAT2 | 1.214388744 | 0.000018 | 0.000136851 |
| NAT1 | 1.121579352 | 0.00000058 | 0.00000744 |
| TYMP | 1.581519236 | 2.82E-14 | 5.37E-12 |
| ASNS | -1.090645553 | 0.000000609 | 0.00000776 |
| AOC3 | 1.126026626 | 0.00000421 | 0.0000397 |
| AOC2 | 1.595746058 | 3.12E-11 | 1.79E-09 |
| CHDH | -1.020677759 | 1.51E-08 | 0.000000344 |
| CBS | -1.063285542 | 0.0000302 | 0.000212444 |
| CTH | -1.676694662 | 1.14E-21 | 3.27E-18 |
| SDSL | 1.495557671 | 0.00000297 | 0.0000296 |
| PRDM16 | -1.538352866 | 4.14E-08 | 0.000000799 |
| CKMT2 | 1.022864146 | 0.0000658 | 0.000412426 |
| P4HA3 | 1.997472518 | 1.06E-09 | 3.52E-08 |
| TPO | 1.08263082 | 0.0000341 | 0.000236197 |
| TDO2 | 1.000836689 | 0.000923903 | 0.003785501 |
| KYNU | 1.949231346 | 0.000000179 | 0.00000278 |
| INMT | 1.232955855 | 0.00000382 | 0.0000367 |
| MARS2 | 1.161622831 | 2.94E-08 | 0.000000601 |
| ANPEP | 2.697330382 | 1.11E-11 | 7.88E-10 |
| HPGDS | 1.564106672 | 0.00000307 | 0.0000305 |
| GPX7 | 1.55710045 | 6.67E-12 | 5.21E-10 |
| GPX3 | -1.794827967 | 1.05E-09 | 3.48E-08 |
| GPX8 | 1.146587448 | 0.000000083 | 0.00000145 |
| DOLK | 1.068591612 | 5.31E-08 | 0.00000099 |
| OSTC | 1.210219723 | 0.0000123 | 0.0000987 |
| ST6GAL2 | 3.023666351 | 1.51E-11 | 1.02E-09 |
| GALNT5 | 1.01306635 | 2.84E-10 | 1.16E-08 |
| GALNT14 | 1.448192648 | 0.000000728 | 0.00000905 |
| GALNT16 | 1.006657023 | 0.000661079 | 0.002865036 |
| GALNT1 | 1.054827719 | 3.4E-12 | 2.86E-10 |
| GALNT7 | 1.055191423 | 1.22E-08 | 0.000000285 |
| MFNG | 1.379565014 | 0.000130566 | 0.000735747 |
| HYAL2 | 1.036601391 | 0.000000286 | 0.00000409 |
| ARSB | 1.201534726 | 4.78E-13 | 5.57E-11 |
| HPSE | 1.105705074 | 0.000334493 | 0.001617776 |
| CHST13 | 1.781563891 | 0.000000253 | 0.00000371 |
| B3GNT2 | 1.108957396 | 0.0000123 | 0.000099 |
| CHST2 | 1.443573001 | 2.93E-11 | 1.7E-09 |
| EXTL2 | 1.386072689 | 0.000000617 | 0.00000785 |
| HS3ST3A1 | -1.3112123 | 0.00000406 | 0.0000386 |
| PLPP4 | 1.738725736 | 0.000000902 | 0.0000108 |
| DGKI | 1.320467866 | 0.000000515 | 0.00000675 |
| MOGAT1 | -1.485927562 | 0.000000271 | 0.00000392 |
| PIK3CG | 1.669237569 | 0.0000121 | 0.0000975 |
| PLCB2 | 1.874150072 | 4.71E-08 | 0.000000896 |
| PIGA | -1.161714992 | 1.22E-12 | 1.2E-10 |
| PLD1 | 1.081745857 | 1.67E-08 | 0.000000374 |
| PLA2G5 | 1.333356227 | 0.00000361 | 0.000035 |
| PNPLA7 | -1.147304326 | 5.04E-09 | 0.000000135 |
| AGPS | 1.001962124 | 2.55E-08 | 0.000000533 |
| ENPP6 | 1.303827841 | 0.00000156 | 0.0000171 |
| ENPP2 | 1.227253983 | 2.33E-08 | 0.000000496 |
| PTGES | 1.762309404 | 6.12E-09 | 0.00000016 |
| PRXL2B | 1.244925624 | 5.75E-10 | 2.08E-08 |
| TBXAS1 | 1.109608721 | 0.0000285 | 0.000202326 |
| ALOX5 | 1.325071279 | 0.0000902 | 0.000541344 |
| CYP4F3 | 1.296616016 | 0.000142009 | 0.000787805 |
| SGMS2 | 1.029451456 | 0.000000205 | 0.00000311 |
| FUT3 | -1.455442084 | 3.89E-08 | 0.000000757 |
| ABO | 1.057438296 | 0.000702239 | 0.003009383 |
| GCNT2 | 1.062532396 | 0.000594706 | 0.002631414 |
| ST6GALNAC5 | 3.639672688 | 6.98E-20 | 1.06E-16 |
| LDHD | -1.14074043 | 3.45E-11 | 1.94E-09 |
| ACSM5 | 1.107269176 | 0.0000114 | 0.0000932 |
| ALDH1L1 | -1.851219363 | 2.05E-11 | 1.31E-09 |
| ALPL | 2.397610676 | 0.000000492 | 0.00000649 |
| ACP5 | 1.212980021 | 0.0000969 | 0.00057424 |
| QPRT | 1.242063059 | 0.0000132 | 0.000104853 |
| CD38 | 1.127947824 | 0.000991513 | 0.004021447 |
| PANK1 | 1.047206483 | 0.000000603 | 0.00000769 |
| VNN2 | 1.252389808 | 0.0000289 | 0.000204626 |
| MOCOS | 1.121305435 | 3.62E-09 | 0.000000101 |
| DHRS3 | -1.332924042 | 1.15E-18 | 1.06E-15 |
| CYP2W1 | -1.274744218 | 0.0000268 | 0.000192094 |
| HEPH | 1.501240553 | 0.00000014 | 0.00000224 |
| CA1 | 1.355519827 | 0.002633941 | 0.009164686 |
| CA12 | 1.863487212 | 0.000169886 | 0.000917738 |
| SQOR | 1.223940033 | 0.00000166 | 0.000018 |

| **Table S5** **Differential expression of 25 OA-related metabolic genes in bulk RNA-seq data from OA cartilage** | | | |
| --- | --- | --- | --- |
| **Genes** | **logFC** | ***p*.Value** | **adj.*p*.Val** |
| HK3 | 1.541972476 | 0.0000707 | 0.000438437 |
| FBP1 | 1.13489171 | 0.000147679 | 0.000813924 |
| LIPA | 1.785267726 | 3.85E-13 | 4.63E-11 |
| GUCY1B1 | 1.248038732 | 0.0000762 | 0.000468098 |
| PDE3A | 1.435261491 | 0.00000114 | 0.0000131 |
| KYNU | 1.949231346 | 0.000000179 | 0.00000278 |
| ANPEP | 2.697330382 | 1.11E-11 | 7.88E-10 |
| HPGDS | 1.564106672 | 0.00000307 | 0.0000305 |
| GPX7 | 1.55710045 | 6.67E-12 | 5.21E-10 |
| ST6GAL2 | 3.023666351 | 1.51E-11 | 1.02E-09 |
| GALNT14 | 1.448192648 | 0.000000728 | 0.00000905 |
| MFNG | 1.379565014 | 0.000130566 | 0.000735747 |
| HPSE | 1.105705074 | 0.000334493 | 0.001617776 |
| HS3ST3A1 | -1.3112123 | 0.00000406 | 0.0000386 |
| PLPP4 | 1.738725736 | 0.000000902 | 0.0000108 |
| PIK3CG | 1.669237569 | 0.0000121 | 0.0000975 |
| PLCB2 | 1.874150072 | 4.71E-08 | 0.000000896 |
| PLA2G5 | 1.333356227 | 0.00000361 | 0.000035 |
| TBXAS1 | 1.109608721 | 0.0000285 | 0.000202326 |
| ALOX5 | 1.325071279 | 0.0000902 | 0.000541344 |
| ALPL | 2.397610676 | 0.000000492 | 0.00000649 |
| ACP5 | 1.212980021 | 0.0000969 | 0.00057424 |
| QPRT | 1.242063059 | 0.0000132 | 0.000104853 |
| HEPH | 1.501240553 | 0.00000014 | 0.00000224 |
| CA12 | 1.863487212 | 0.000169886 | 0.000917738 |

**Table S6 Maker genes of six clusters**

|  | ***p*.Val** | **avg_log2FC** | **pct.1** | **pct.2** | **adj*.p.*Val** | **cluster** |
| --- | --- | --- | --- | --- | --- | --- |
| CENPU | 0 | 0.75924 | 0.327 | 0.013 | 0 | CPCs |
| STMN1 | 2.0472E-281 | 2.537606 | 0.507 | 0.038 | 6.9E-277 | CPCs |
| BIRC5 | 0 | 0.794038 | 0.263 | 0.002 | 0 | CPCs |
| TOP2A | 0 | 1.209549 | 0.395 | 0.002 | 0 | CPCs |
| CDK1 | 0 | 1.138552 | 0.351 | 0.002 | 0 | CPCs |
| UBE2C | 0 | 1.335961 | 0.356 | 0.004 | 0 | CPCs |
| CENPF | 0 | 1.485838 | 0.405 | 0.008 | 0 | CPCs |
| ASPM | 0 | 0.924101 | 0.298 | 0.002 | 0 | CPCs |
| SAMSN1 | 0 | 1.331279 | 0.215 | 0.001 | 0 | CPCs |
| PBK | 0 | 0.60339 | 0.263 | 0 | 0 | CPCs |
| NUSAP1 | 0 | 1.726375 | 0.429 | 0.016 | 0 | CPCs |
| SPC25 | 0 | 0.443871 | 0.19 | 0 | 0 | CPCs |
| TPX2 | 0 | 1.068328 | 0.322 | 0.007 | 0 | CPCs |
| PTPRC | 0 | 1.91614 | 0.21 | 0.002 | 0 | CPCs |
| LCP1 | 0 | 1.954494 | 0.278 | 0.005 | 0 | CPCs |
| HBA2 | 0 | 6.274321 | 0.704 | 0.175 | 0 | HBB+ cells |
| HBA1 | 0 | 5.793263 | 0.663 | 0.122 | 0 | HBB+ cells |
| HBB | 0 | 6.958233 | 0.784 | 0.316 | 0 | HBB+ cells |
| HBM | 0 | 1.759521 | 0.153 | 0.008 | 0 | HBB+ cells |
| SNCA | 0 | 1.512339 | 0.182 | 0.036 | 0 | HBB+ cells |
| ALAS2 | 0 | 1.082474 | 0.112 | 0.005 | 0 | HBB+ cells |
| FTH1 | 0 | 0.565353 | 1 | 0.995 | 0 | HBB+ cells |
| MT2A | 0 | 0.272599 | 1 | 0.992 | 0 | HBB+ cells |
| FTL | 0 | 1.024773 | 0.999 | 0.985 | 0 | HBB+ cells |
| MGP | 0 | 0.295126 | 1 | 0.987 | 0 | HBB+ cells |
| S100A6 | 0 | 0.547556 | 1 | 0.98 | 0 | HBB+ cells |
| MSMP1 | 5.25E-178 | 1.332469004 | 0.065 | 0.268 | 1.76E-173 | HBB+ cells |
| RPS12 | 2E-203 | 0.302226 | 0.995 | 0.975 | 6.7E-199 | HBB+ cells |
| SLC25A37 | 1.58E-14 | 1.122838174 | 0.519 | 0.762 | 5.29E-10 | HBB+ cells |
| RPLP1 | 2.3E-174 | 0.262033 | 0.998 | 0.982 | 7.7E-170 | HBB+ cells |
| MMP3 | 2E-305 | 0.658943 | 0.98 | 0.959 | 6.7E-301 | preHTCs |
| COL2A1 | 0 | 0.38281 | 0.998 | 0.928 | 0 | preHTCs |
| COL6A1 | 1.31E-271 | 0.845829825 | 0.857 | 0.86 | 4.4E-267 | preHTCs |
| TNFRSF11B | 0 | 0.591298 | 0.994 | 0.908 | 0 | preHTCs |
| CLU | 0 | 0.421087 | 0.998 | 0.973 | 0 | preHTCs |
| DCN | 3.9E-271 | 0.371559 | 0.998 | 0.973 | 1.3E-266 | preHTCs |
| SERPINE2 | 2.2E-254 | 0.524785 | 0.946 | 0.919 | 7.5E-250 | preHTCs |
| MT2A | 0 | 0.272599 | 1 | 0.992 | 0 | preHTCs |
| MALAT1 | 0 | 0.254799 | 0.996 | 0.97 | 0 | preHTCs |
| FTH1 | 0 | 0.565353 | 1 | 0.995 | 0 | preHTCs |
| C2ORF40 | 0 | 0.718654 | 0.999 | 0.959 | 0 | preHTCs |
| MGP | 0 | 0.295126 | 1 | 0.987 | 0 | preHTCs |
| COMP | 0 | 0.280765 | 0.998 | 0.946 | 0 | preHTCs |
| INHBA | 0 | 0.417029 | 0.989 | 0.889 | 0 | preHTCs |
| MT-ATP6 | 0 | 1.945534 | 0.925 | 0.913 | 0 | preHTCs |
| FOSB | 0 | 1.211112809 | 0.386 | 0.113 | 0 | HomCs |
| DNAJB4 | 0 | 0.939635 | 0.434 | 0.2 | 0 | HomCs |
| SNHG12 | 0 | 1.287219 | 0.449 | 0.222 | 0 | HomCs |
| HSPA6 | 0 | 3.509594 | 0.4 | 0.042 | 0 | HomCs |
| HSPB1 | 0 | 2.534843 | 0.574 | 0.237 | 0 | HomCs |
| HSPA1B | 0 | 2.910217 | 0.543 | 0.246 | 0 | HomCs |
| DNAJB1 | 0 | 2.335356 | 0.59 | 0.356 | 0 | HomCs |
| HSPA1A | 0 | 3.648057 | 0.619 | 0.389 | 0 | HomCs |
| BAG3 | 0 | 1.083579 | 0.508 | 0.291 | 0 | HomCs |
| NDUFA4L2 | 0 | 1.321493 | 0.886 | 0.724 | 0 | HomCs |
| AL118516.1 | 0 | 1.058348 | 0.408 | 0.159 | 0 | HomCs |
| LRIF1 | 0 | 1.023201 | 0.397 | 0.152 | 0 | HomCs |
| THUMPD3-AS1 | 0 | 1.121693 | 0.501 | 0.302 | 0 | HomCs |
| ITM2B | 0 | 0.393708 | 0.971 | 0.898 | 0 | HomCs |
| SOD3 | 0 | 0.864626 | 0.956 | 0.878 | 0 | HomCs |
| SQSTM1 | 0 | 0.788877666 | 0.955 | 0.849 | 0 | ECs |
| ASNS | 0 | 0.330469502 | 0.481 | 0.362 | 0 | ECs |
| ARHGAP21 | 0 | 0.322213786 | 0.644 | 0.54 | 0 | ECs |
| NOP16 | 2.4141E-270 | 0.294975 | 0.406 | 0.297 | 8.1E-266 | ECs |
| FOSL1 | 0 | 0.722838 | 0.52 | 0.4 | 0 | ECs |
| RPS27 | 0 | 0.737575 | 0.976 | 0.935 | 0 | ECs |
| RPL39 | 0 | 0.291082 | 0.992 | 0.933 | 0 | ECs |
| NACA | 0 | 0.260223 | 0.985 | 0.916 | 0 | ECs |
| RPL24 | 0 | 0.598308 | 0.978 | 0.931 | 0 | ECs |
| RPS13 | 8E-272 | 0.413292 | 0.984 | 0.942 | 2.7E-267 | ECs |
| RPL37 | 2.1E-281 | 0.508058 | 0.967 | 0.92 | 7.2E-277 | ECs |
| RPS3A | 0 | 0.561178 | 0.985 | 0.949 | 0 | ECs |
| RPS15 | 0 | 0.253075 | 0.99 | 0.931 | 0 | ECs |
| RPS28 | 0 | 0.250093 | 0.993 | 0.937 | 0 | ECs |
| RPL34 | 0 | 0.664111 | 0.977 | 0.93 | 0 | ECs |
| CLCF1 | 0 | 0.287640277 | 0.548 | 0.276 | 0 | ProCs |
| CHAD | 0 | 0.767165 | 0.918 | 0.505 | 0 | ProCs |
| ASNS | 0 | 0.722261 | 0.989 | 0.725 | 0 | ProCs |
| P3H2 | 0 | 0.614698 | 0.941 | 0.528 | 0 | ProCs |
| S100A1 | 0 | 0.761532 | 0.977 | 0.687 | 0 | ProCs |
| COL9A3 | 0 | 0.369677 | 0.968 | 0.666 | 0 | ProCs |
| PMEPA1 | 0 | 0.668567 | 0.912 | 0.569 | 0 | ProCs |
| PDLIM4 | 0 | 0.645718 | 0.93 | 0.628 | 0 | ProCs |
| FGFBP2 | 0 | 0.977643 | 0.989 | 0.753 | 0 | ProCs |
| PLOD2 | 0 | 0.409158 | 0.961 | 0.711 | 0 | ProCs |
| COL6A3 | 0 | 0.466106 | 0.906 | 0.536 | 0 | ProCs |
| SNORC | 0 | 0.8603 | 0.996 | 0.808 | 0 | ProCs |
| COL6A1 | 0 | 0.456821 | 0.988 | 0.795 | 0 | ProCs |
| RGS3 | 0 | 0.58047 | 0.907 | 0.59 | 0 | ProCs |
| COL2A1 | 0 | 0.702557 | 0.954 | 0.672 | 0 | ProCs |

| **Table S7** **Functional analysis of mark genes in each cell cluster** | | | | |
| --- | --- | --- | --- | --- |
| **Cluster** | **Description** | **GeneRatio** | **pvalue** | **Annotation** |
| 0 | collagen metabolic process | 0.050 | 3.50E-06 | ECs |
| 0 | unsaturated fatty acid metabolic process | 0.050 | 6.44E-06 | ECs |
| 0 | C21-steroid hormone metabolic process | 0.025 | 2.71E-04 | ECs |
| 0 | glycoside metabolic process | 0.019 | 7.24E-04 | ECs |
| 0 | fatty acid metabolic process | 0.056 | 7.61E-03 | ECs |
| 1 | positive regulation of epithelial cell migration | 0.034 | 3.00E-03 | ProCs |
| 1 | epithelium migration | 0.049 | 7.58E-03 | ProCs |
| 1 | endothelium development | 0.025 | 1.93E-02 | ProCs |
| 1 | endothelial cell differentiation | 0.020 | 4.51E-02 | ProCs |
| 1 | positive regulation of blood vessel endothelial cell migration | 0.015 | 4.87E-02 | ProCs |
| 2 | regulation of transcription from RNA polymerase II promoter in response to stress | 0.018 | 4.84E-06 | HomC |
| 2 | regulation of DNA-templated transcription in response to stress | 0.018 | 1.56E-05 | HomC |
| 2 | response to starvation | 0.038 | 6.77E-05 | HomC |
| 2 | embryonic organ development | 0.053 | 7.19E-04 | HomC |
| 2 | regulation of mRNA metabolic process | 0.045 | 7.69E-04 | HomC |
| 3 | extracellular matrix organization | 0.165 | 1.91E-20 | preHTC |
| 3 | extracellular structure organization | 0.165 | 2.08E-20 | preHTC |
| 3 | collagen fibril organization | 0.068 | 7.79E-13 | preHTC |
| 3 | connective tissue development | 0.108 | 2.30E-11 | preHTC |
| 3 | collagen metabolic process | 0.051 | 6.58E-07 | preHTC |
| 4 | hydrogen peroxide catabolic process | 0.217 | 2.81E-10 | HBB^+^ cells |
| 4 | oxygen transport | 0.174 | 2.27E-09 | HBB^+^ cells |
| 4 | hydrogen peroxide metabolic process | 0.217 | 5.14E-09 | HBB^+^ cells |
| 4 | gas transport | 0.174 | 1.21E-08 | HBB^+^ cells |
| 4 | carbon dioxide transport | 0.130 | 7.13E-07 | HBB^+^ cells |
| 5 | chromosome segregation | 0.140 | 3.78E-36 | CPCs |
| 5 | mitotic cell cycle phase transition | 0.134 | 1.36E-30 | CPCs |
| 5 | nuclear chromosome segregation | 0.111 | 3.39E-30 | CPCs |
| 5 | DNA replication | 0.073 | 7.98E-16 | CPCs |
| 5 | cell cycle G1/S phase transition | 0.053 | 9.48E-09 | CPCs |
